# Activation of the integrated stress response rewires cardiac metabolism in Barth syndrome

**DOI:** 10.1007/s00395-023-01017-x

**Published:** 2023-11-06

**Authors:** Ilona Kutschka, Edoardo Bertero, Christina Wasmus, Ke Xiao, Lifeng Yang, Xinyu Chen, Yasuhiro Oshima, Marcus Fischer, Manuela Erk, Berkan Arslan, Lin Alhasan, Daria Grosser, Katharina J. Ermer, Alexander Nickel, Michael Kohlhaas, Hanna Eberl, Sabine Rebs, Katrin Streckfuss-Bömeke, Werner Schmitz, Peter Rehling, Thomas Thum, Takahiro Higuchi, Joshua Rabinowitz, Christoph Maack, Jan Dudek

**Affiliations:** 1 Department of Translational Research, Comprehensive Heart Failure Center (CHFC), University Clinic Würzburg, Am Schwarzenberg 15, Haus A15, 97078 Würzburg, Germany; 2 Department of Internal Medicine, University of Genova, Genoa, Italy; 3 Cardiovascular Disease Unit, IRCCS Ospedale Policlinico San Martino – Italian IRCCS Cardiology Network, Genoa, Italy; 4 Institute of Molecular and Translational Therapeutic Strategies, Hannover Medical School, Carl-Neuberg-Str. 1, 30625 Hannover, Germany; 5 Fraunhofer Institute for Toxicology and Experimental Medicine (ITEM), Nikolai-Fuchs-Straße 1, 30625 Hannover, Germany; 6 Shanghai Institute of Nutrition and Health, Chinese Academy of Sciences, 320 Yueyang Rd, Shanghai, 200031 China; 7 Department of Nuclear Medicine, University Clinic Würzburg, Oberdürrbacher Strasse 6, 97080 Würzburg, Germany; 8 Division of Pediatric Cardiology and Intensive Care, University Hospital LMU Munich, Marchioninistr. 15, 81377 Munich, Germany; 9 Department for Pharmacology and Toxicology, University of Würzburg, Versbacher Strasse 9, 97078 Würzburg, Germany; 10 Clinic for Cardiology and Pneumology, Georg-August University Göttingen and DZHK (German Center for Cardiovascular Research), Partner Site, Göttingen, Germany; 11 Department of Biochemistry and Molecular Biology, University of Würzburg, Am Hubland, 97074 Würzburg, Germany; 12 University Göttingen, Institute of Biochemistry and Molecular Cell Biology, Humboldtallee 23, 37072 Göttingen, Germany; 13 Cluster of Excellence “Multiscale Bioimaging: From Molecular Machines to Networks of Excitable Cells” (MBExC), University of Göttingen, Göttingen, Germany; 14 Rebirth Center for Translational Regenerative Medicine, Hannover Medical School, Carl-Neuberg-Str. 1, 30625 Hannover, Germany; 15 Lewis-Sigler Institute for Integrative Genomics, Princeton University, Princeton, NJ 08544 USA; 16 Medical Clinic I, University Clinic Würzburg, Würzburg, Germany

**Keywords:** Barth syndrome, Mitochondria, Fatty acid oxidation, Metabolism, Oxidative stress, Amino acid

## Abstract

Barth Syndrome (BTHS) is an inherited cardiomyopathy caused by defects in the mitochondrial transacylase TAFAZZIN (*Taz*), required for the synthesis of the phospholipid cardiolipin. BTHS is characterized by heart failure, increased propensity for arrhythmias and a blunted inotropic reserve. Defects in Ca^2+^-induced Krebs cycle activation contribute to these functional defects, but despite oxidation of pyridine nucleotides, no oxidative stress developed in the heart. Here, we investigated how retrograde signaling pathways orchestrate metabolic rewiring to compensate for mitochondrial defects. In mice with an inducible knockdown (KD) of TAFAZZIN, and in induced pluripotent stem cell-derived cardiac myocytes, mitochondrial uptake and oxidation of fatty acids was strongly decreased, while glucose uptake was increased. Unbiased transcriptomic analyses revealed that the activation of the eIF2α/ATF4 axis of the integrated stress response upregulates one-carbon metabolism, which diverts glycolytic intermediates towards the biosynthesis of serine and fuels the biosynthesis of glutathione. In addition, strong upregulation of the glutamate/cystine antiporter xCT increases cardiac cystine import required for glutathione synthesis. Increased glutamate uptake facilitates anaplerotic replenishment of the Krebs cycle, sustaining energy production and antioxidative pathways. These data indicate that ATF4-driven rewiring of metabolism compensates for defects in mitochondrial uptake of fatty acids to sustain energy production and antioxidation.

**Supplementary Information:**

The online version contains supplementary material available at 10.1007/s00395-023-01017-x.

## Introduction

Mitochondrial disorders are extremely heterogeneous and often involve a variable degree of neurological, muscular, and cardiac manifestations. Since the heart relies primarily on mitochondrial oxidative metabolism to sustain the energy-demanding processes of cardiac contraction and relaxation, impairment in oxidative phosphorylation is a central driver of cardiomyopathy. However, little is known about the consequences of mitochondrial dysfunction on metabolic pathways in cardiac myocytes. Barth syndrome (BTHS) is a mitochondrial disorder characterized by early onset of cardiomyopathy, skeletal muscle myopathy, immune defects, and growth retardation [4]. The pathogenic mutation resides in the X-chromosome-linked gene encoding the mitochondrial transacylase tafazzin (*Taz*), which catalyzes maturation of the mitochondrial phospholipid cardiolipin (CL) [9]. Loss of *Taz* function depletes mature forms of CL, alters its acylation pattern, and increases abundance of the CL precursor monolysocardiolipin [43]. In BTHS, the abnormal lipid composition of the inner mitochondrial membrane affects mitochondrial morphology and alters the structural organization of respiratory chain “supercomplexes” (respirasomes), thereby impairing ATP production [26, 37]. Inefficient electron transfer can lead to excessive reactive oxygen species (ROS) formation at the respiratory chain, imposing an increased requirement for reducing equivalents to sustain ROS elimination [18, 85]. Besides the respiratory chain, also other macromolecular complexes located in the inner mitochondrial membrane are affected by CL depletion, including succinate dehydrogenase [27], pyruvate dehydrogenase (PDH) [56] and the mitochondrial Ca^2+^ uniporter (MCU) [6, 34].

Ca^2+^ taken up via the MCU is required to stimulate rate-limiting dehydrogenases of the Krebs cycle during cardiac workload transitions. The Krebs cycle produces reduced nicotinamide adenine dinucleotide (NADH) not only for ATP production, but also reduced nicotinamide adenine dinucleotide phosphate (NADPH) to eliminate hydrogen peroxide (H_2_O_2_) via the glutathione peroxidase (GPX) and the thioredoxin/peroxiredoxin systems [5]. Defects in cytosolic and mitochondrial Ca^2+^ handling contribute to energetic deficit and oxidative stress in heart failure [5, 51]. We reported previously that mice with global short hairpin-mediated knockdown of *Taz* (*Taz*-KD) show alterations in CL, typical for BTHS patients [27]. Furthermore, these mice develop heart failure with preserved ejection fraction, a blunted inotropic response to β-adrenergic stimulation and predisposition to arrhythmias [6], closely resembling the clinical phenotype of the majority of patients with BTHS cardiomyopathy who do not deteriorate towards death or transplantation [20, 74, 75]. Conversely, in the latter patients, a phenotype of dilated cardiomyopathy develops [20], which is reflected more closely by a mouse model with a complete knock-out of *Taz* with severe systolic dysfunction [58, 86]. In contrast to this, more severe form of BTHS cardiomyopathy, in which increased mitochondrial ROS were observed [58], we [6] and others [35] did neither observe an increase in mitochondrial ROS in isolated mitochondria, isolated cardiac myocytes nor in vivo in *Taz*-KD hearts. This was against our expectations, since we discovered severe MCU downregulation in *Taz*-KD hearts, which compromised Krebs cycle-dependent NAD(P)H regeneration during cardiac workload transitions, which based on our previous work [51], should lead to an increase in mitochondrial emission of H_2_O_2_. Despite this lack of increase in mitochondrial ROS, mitochondrial NADH and NADPH oxidation contributed to cellular arrhythmias and slowing of electrical conduction [6]. We reasoned that upregulated protein expression of H_2_O_2_-eliminating GPX1 might prevent oxidative stress in *Taz*-KD hearts [6]. However, the mechanisms that upregulate these proteins and alter metabolic pathways that fuel these antioxidative systems in BTHS are completely unknown.

**Fig. 1:**
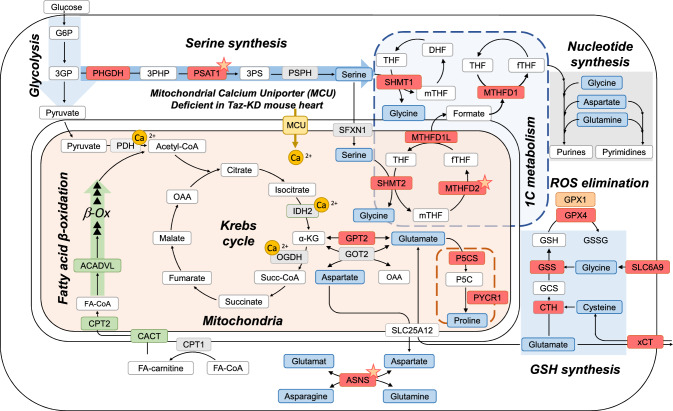
Metabolic rewiring in BTHS. Summary of changes in gene expression pattern in cardiac samples from *Taz*-KD mice compared to WT. Red indicates significant upregulation in transcriptomic data described in Fig. 3, genes with a published AARE element in their promoter are indicated with a star. Green indicates significant downregulation, amino acids are indicated in blue. (Ca^2+^ calcium, *OAA* ocaloacetate, *GCS* γ-glutamylcysteine, *P5C* pyroline-5-carboxylate, *G6P* glucose-6-phosphate, *3GP* 3-phosphoglycerate, *3PHP* 3-phosphohydroxypyruvate, *3PS* 3-phosphoserine)

The transcriptional response to organelle dysfunction is typically orchestrated by retrograde signaling pathways that maintain cellular homeostasis in response to diverse environmental and pathological conditions. The integrated stress response (ISR) is an evolutionarily conserved signaling pathway, activated in response to mitochondrial dysfunction, altered proteostasis, nutrient deprivation, and oxidative stress [21, 48, 64, 80]. Cellular stressors trigger the ISR by activating the specialized sensor kinases GCN2, PKR, HRI and PERK that catalyze phosphorylation of the eukaryotic initiation factor 2α (eIF2α), a component of the initiator complex of cytosolic protein translation [23, 42, 70]. eIF2α phosphorylation leads to a general reduction in ribosomal translation and at the same time, triggers the preferred translation of a selected set of genes, such as the basic region-leucine zipper activating transcription factor 4 (ATF4) [40]. Stress-induced activation of ATF4 induces expression of genes involved in serine biosynthesis and one-carbon (1C) metabolism [42] by its affinity for the amino acid response element (AARE) in the promoters of responsive genes (Fig. 1) [48, 64, 80]. This 1C metabolism supports numerous cellular processes, including purine and thymidine biosynthesis, and is pivotal to amino acid homeostasis [25]. Furthermore, 1C metabolism plays a central role in maintaining cellular antioxidant defense: conversion of serine to glycine fuels glutathione (GSH) synthesis, and reduction of NADPH by the cytosolic and mitochondrial methylenetetrahydrofolate dehydrogenase isozymes (MTHFD1 and MTHFD2, respectively) can impact the NADPH/NADP^+^ ratio (Fig. 1) [29]. However, the role of ATF4 signaling and its implication on cardiac metabolism in the heart is currently only incompletely resolved.

In BTHS, exercise intolerance is closely associated with defects in cardio-skeletal substrate metabolism. During sub-maximal exercise, the physiological increase in total FA oxidation rate is blunted in patients with BTHS, which was associated with decreased phosphocreatine (PCr) to ATP ratios in skeletal muscles and the heart [12]. Furthermore, reduced myocardial FA extraction and uptake at rest correlated with cardiac dysfunction in BTHS patients [13]. In contrast, myocardial glucose extraction and utilization are increased in BTHS patients, while the exercise-induced increase in total glucose oxidation is blunted and lactate accumulation increased [12, 13]. These data indicate that substantial metabolic rewiring occurs in hearts of BTHS patients, with a substrate switch away from FA towards glucose utilization, despite compromised glucose oxidation in mitochondria. However, the underlying mechanisms of this metabolic switch are currently unresolved.

Here, we demonstrate that metabolic alterations in BTHS are associated with an activation of the ATF4 signaling cascade to increase cystine uptake, serine to glycine conversion and 1C metabolism to support GSH biosynthesis. Furthermore, ATF4 controls glutamate metabolism, which on one hand serves to maintain anaplerotic refueling of intermediate metabolism to secure energy conversion, and on the other hand, to facilitate cystine uptake via the xCT system for the synthesis of glutathione. Together, these results identify a comprehensive rewiring of cardiac metabolism in BTHS that compensates for the primary mitochondrial defects induced by *Taz* deficiency and altered CL remodeling.

## Results

### Uptake and utilization of fatty acids is impaired in tafazzin-deficient hearts and iPSC-derived cardiac myocytes

**Fig. 2:**
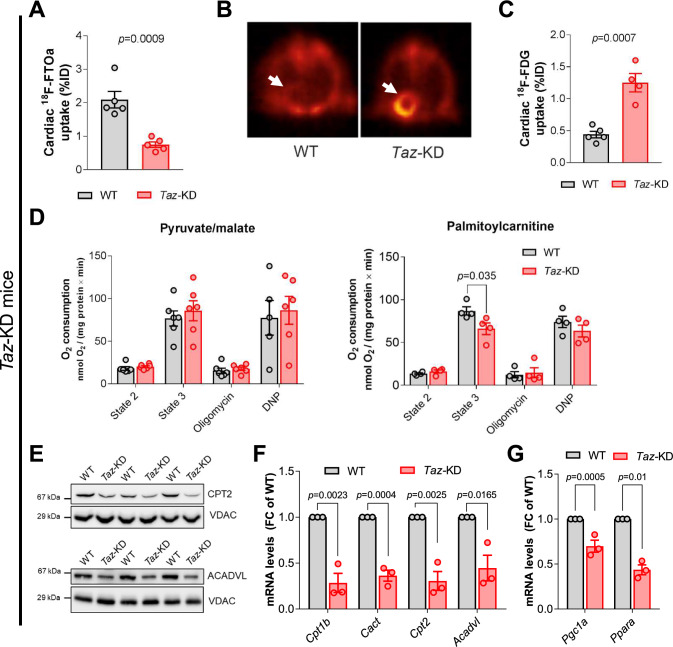
Uptake and utilization of fatty acids is impaired in tafazzin-deficient mouse hearts. **A** Cardiac ^18^F-FTOa uptake normalized to blood levels in WT and *Taz*-KD mice (12 weeks old). *n* = 5 per genotype. **B** Representative PET-CT images of cardiac ^18^F-FDG uptake in WT and *Taz*-KD hearts of 12-week-old mice. **C** Quantification of cardiac ^18^F-FDG uptake normalized to lung uptake in WT and *Taz*-KD mice. *n* = 5 per genotype*.* **D** Oxygen consumption rate (OCR) of isolated cardiac mitochondria from 20-week-old WT and *Taz*-KD mice supplied with pyruvate and malate (left panel, *n* = 6 per genotype) or palmitoylcarnitine (right panel, *n* = 4 per genotype) in absence (state 2) and presence (state 3) of ADP (1 mM) before adding oligomycin and 2,4-dinitrophenol (DNP). **E** Western blot analysis of indicated proteins in isolated mitochondria. **F** Cardiac mRNA levels of indicated genes normalized to the mtDNA-encoded ribosomal RNA mS12. *n* = 3 per genotype. **G** Cardiac mRNA levels of *Pgc1a* and *Ppara* normalized to *Gapdh.* *n* = 3 per genotype. Data are mean ± SEM; *n*-numbers are numbers of hearts or animals. Statistical significance was determined with unpaired Student *t*-test for panels A, C, F, G, and by two-way ANOVA followed by Bonferroni post-test for panel D

Myocardial glucose utilization is increased and FA uptake is decreased in patients with BTHS compared to healthy subjects [13]. In agreement with this, we observed higher cardiac glucose uptake and lower FA uptake and integration into β-oxidation in *Taz*-KD mice, as assessed in vivo by positron emission tomography with fluorodeoxyglucose (^18^F-FDG) and the metabolically trapped FA radiotracer ^18^F-FTOa, respectively (Fig. 2A–C) [66]. Changes in substrate utilization occurred despite unchanged blood glucose levels, heart weight, and gastrocnemius muscle weight. Body weight of *Taz*-KD mice was lower than of WT littermates (Supplementary Fig. 1A–D), as shown previously [6] and in agreement with the clinical phenotype of growth retardation [32]. Increased glucose uptake occurred in absence of an induction of gene expression of glucose transporters. Only a moderate increase in *Glut1* gene expression was detected by qPCR (Supplementary Fig. 1E).

To interrogate whether the shift in substrate uptake reflects changes in mitochondrial oxidative capacity, we determined respiration of isolated cardiac mitochondria in the presence of different substrates. Maximal ADP-stimulated O_2_ consumption was similar in *Taz*-KD and WT isolated cardiac mitochondria respiring on pyruvate and malate, but reduced in *Taz*-KD mitochondria when fueled with palmitoylcarnitine (Fig. 2D) or the short-chain FA octanoylcarnitine (Supplementary Fig. 1F), which still requires the carnitine palmitoyl transferase 2, CPT2 (Fig. 1) for the transport across the mitochondrial membranes [6]. As an underlying mechanism for defective myocardial FA uptake and oxidation, we observed reduced protein levels of CPT2 and acyl-CoA dehydrogenase very long-chain ACADVL (Fig. 2E, Supplementary Fig. 1G). Reduced protein levels are consistent with reduced gene expression of *Cpt1b*, *Cpt2*, *Acadvl* and the carnitine/acylcarnitine carrier *Cact* (Fig. 2F). The trimethyllysine hydroxylase (TMLHE), catalyzing the first step in the carnitine biosynthesis pathway, was reduced on protein and mRNA levels in *Taz*-KD mouse hearts (Supplementary Fig. 1H, I). Also, gene expression of the acyl-CoA synthase providing the essential cofactor Coenzyme A (CoA) was reduced (Supplementary Fig. 1 J). As a potential upstream mechanism, we observed downregulation of the major transcription factors of FA metabolism, i.e., PPARα (gene: *Ppara*) and PGC-1α (gene: *Pgc1a*, Fig. 2G). Taken together, the *Taz*-KD model recapitulates the shift in myocardial substrate utilization observed in BTHS patients, with downregulation of cardiac FA uptake and oxidation, but upregulation of glucose uptake [13].

Increased glucose uptake may increase the burden of O-linked residues of N-acetylglucosamine on cardiac proteins in BTHS. However, we did not observe any changes in the O-GlcNAcylation in cardiac lysates of *Taz*-KD mice (Supplementary Fig. 1K). Mitophagy in *Taz*-deficient mouse models seems to be differently regulated, dependent on the mouse background [87, 92]. We, therefore, tested protein expression of the mitophagy sensor PINK1, which accumulated on isolated cardiac mitochondria of *Taz*-KD mice (Supplementary Fig. 1L and  M). As PINK accumulation is suggestive for recognition of dysfunctional mitochondria by the autophagy system, we analyzed the extent of mitochondrial clearance in *Taz*-KD mice. Unchanged processing of the autophagy receptor LC3-I into the activated LC3-II form argues against elevated execution of mitophagy (Supplementary Fig. 1N). These data indicate that PINK is recruited to dysfunctional mitochondria, but that mitophagy is not executed in *Taz*-KD mitochondria, a result comparable to findings in the *Taz* knock-out in C57BL6J and CAST F1 mouse strains [87]. Elevated mRNA levels of the anti-apoptotic factor *Bcl-2* in *Taz*-KD mouse heart tissue indicate that cellular stress is well compensated (Supplementary Fig. 1O).

Next, we analyzed whether metabolic alterations as described in mice are also evident in human cells. We, therefore, exploited differentiated cardiac myocytes derived from induced pluripotent stem cells (iPSC) of a BTHS patient, which we have characterized before (TAZ10, c. 590 G > T, p. Gly197Val) [26, 27]. Respiration of TAZ10 iPSC-CM on palmitate was lower than control iPSC-CM (Supplementary Fig. 2A). Akin to *Taz-*KD hearts, TAZ10 iPSC-CM exhibited lower levels of CPT2 and CPT1b protein (data not shown) as well as reduced gene expression of *CPT1B*, *CACT*, and *ACADVL* compared with control iPSC-CM (Supplementary Fig. 2B). To substantiate the defect in mitochondrial import of FAs, we supplied iPSC-CM with the medium-chain FA dodecanoate covalently bound to the fluorescent dye Bodipy. Bodipy-labeled FAs are stored in lipid droplets (Supplementary Fig. 2C) that are mobilized and transported into mitochondria upon starvation from glucose for 24 h. Manual counting of transport events (Supplementary Fig. 2D) and automated determination of lipid droplet area before and after starvation (Supplementary Fig. 2E) demonstrated a defective mitochondrial uptake of FA in TAZ10 vs. WT iPSC-CM.

Skeletal myopathy is a dominant feature of BTHS and contributes to exercise intolerance and fatigue. Exercise-induced increases in skeletal muscle O_2_ utilization are reduced in BTHS patients [74]. We, therefore, assessed whether changes in substrate utilization are also evident in skeletal muscle in *Taz*-KD mice. In contrast to hearts, uptake of ^18^F-FDG and ^18^F-FTOa under resting conditions were similar in skeletal muscle of *Taz*-KD and WT mice (Supplementary Fig. 3A, B). While maximal respiration of mitochondria isolated from hind-limb muscle of *Taz*-KD mice was comparable to WT when pyruvate and malate were used as substrates, respiration was lower compared to WT in the presence of palmitoylcarnitine and palmitoyl-CoA (Supplementary Fig. 3C–E). Protein levels of CACT and TMLHE, but not CPT2, were lower in *Taz*-KD vs. WT skeletal muscle (Supplementary Fig. 3F). Interestingly, and in contrast to the heart, mRNA levels of *Cpt2*, *Cact*, *Acadvl*, and *Pgc1a* were unchanged in skeletal muscle of *Taz*-KD mice (Supplementary Fig. 3G). Overall, although skeletal muscle uptake and oxidation of FA under resting conditions are not significantly changed in vivo*,* the maximal capacity of FA oxidation is reduced in *Taz*-KD skeletal muscle mitochondria. Such decreased maximal FA oxidation capacity may contribute to the lack of FA oxidation upon exercise in BTHS patients [12].

### Serine and 1C metabolism is activated in *Taz*-KD hearts

**Fig. 3:**
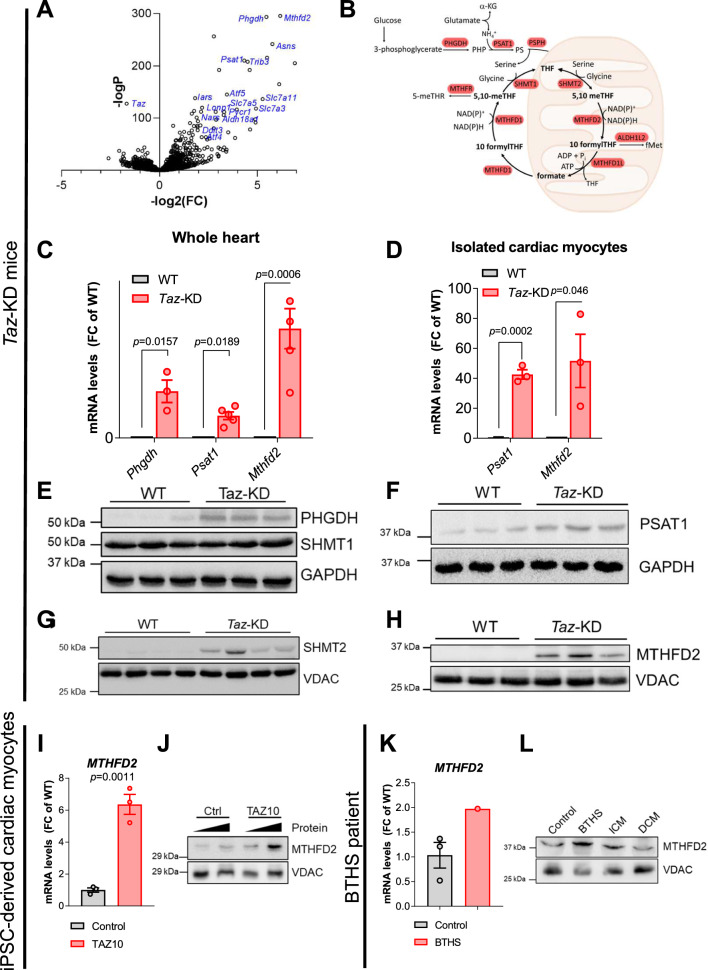
Key enzymes of serine and 1C metabolism are upregulated in BTHS. **A** Volcano plot of log2 fold change versus -log p values of all expressed genes in heart muscle of 20-week-old *Taz*-KD mice in comparison with WT. X axis: log2 transformed fold changes. Y-axis: minus log10 transformed *p* value. *n* = 5 per genotype. **B** Scheme of metabolic pathways with significantly upregulated genes in *Taz*-KD vs. WT hearts highlighted in red. **C** mRNA levels in mouse hearts normalized to mS12 or *Gapdh*, respectively. *n* = 3 per genotype. **D** mRNA levels in cardiac myocytes isolated from 12 week old mice by Langendorff perfusion normalized to mS12. *n* = 3 per genotype. Western blot analysis of indicated proteins in mouse cardiac lysates (**E**, **F**) or isolated mouse cardiac mitochondria (**G, H**). *n* = 3 per genotype. **I** mRNA levels normalized to L28 in iPSC-derived cardiac myocytes. *n* = 3 technical replicates, one representative experiment shown. **J** Western blot analysis in iPSC-derived cardiac myocytes. One representative experiment shown. Samples loaded in two concentrations.** K** mRNA levels normalized to L28 in myocardial samples from one BTHS patient and three controls without heart failure. **L** Western blot analysis in myocardial samples from one healthy control, one BTHS patient, one patient with ischemic heart disease (IHD), and one patient with dilated cardiomyopathy (DCM). Data are mean ± SEM; n-numbers are numbers of hearts or animals for panels A to H and technical replicates in panel I and K; statistical significance was determined with unpaired Student *t*-test

The shift in myocardial substrate utilization points towards an extensive rewiring of cardiac metabolism in the BTHS mouse model. To assess the underlying mechanism of this rewiring, we performed gene expression profiling by RNA sequencing of *Taz*-KD hearts and identified 7.221 differentially expressed genes (adjusted *p* value < 0.05; Supplementary Fig. 4A, [22]). Functional enrichment analysis revealed upregulation of pathways involved in amino acid metabolism (Supplementary Fig. 4B), as well as genes involved in serine biosynthesis and 1C metabolism in *Taz*-KD vs. WT hearts by manual annotation (Fig. 3A). The serine biosynthesis pathway produces serine from the glycolytic intermediate 3-phosphoglycerate via the enzymes phosphoglycerate dehydrogenase (*Phgdh*, log2(FC) = 5.46; *p* < 0.0001) and phosphoserine aminotransferase 1 (*Psat1*, log2(FC) = 4.33; *p* < 0.0001; Fig. 1). Conversion of serine to glycine is the primary source of 1C units for tetrahydrofolate, which in turn serves as a donor of 1C units in a variety of cellular processes, such as purine and thymidine biosynthesis (Figs. 1, 3B) [25]. We replicated RNA sequencing data by quantitative real-time polymerase chain reaction (qPCR) and confirmed marked upregulation of *Phgdh*, *Psat1* and the 1C enzyme methylenetetrahydrofolate dehydrogenase (*Mthfd2*, log2(FC) = 6.17; *p* < 0.0001) in whole hearts (Fig. 3C) and *Psat1 and Mthfd2* specifically in isolated cardiac myocytes (Fig. 3D). Increase in gene expression translates into increased protein levels of serine pathway (PHGDH, PSAT1) and 1C cycle (SHMT2, MTHFD2) enzymes in *Taz*-KD vs. WT hearts (Fig. 3E–H, Supplementary Fig. 5A–D). Interestingly, protein levels of SHMT1 of the cytosolic arm of the 1C metabolism pathway are not strongly upregulated (Fig. 3E), indicating a more prominent upregulation of the mitochondrial arm of the 1C cycle, which is also reflected by the induction of *Shmt2* gene expression, but not of *Shmt1* (Supplementary Fig. 5E, F).

We next elucidated how remodeling of fatty acid oxidation and 1C metabolism interrelate with the onset of the cardiomyopathy phenotype. We have previously delineated the onset of cardiomyopathy at an age of later than 10 weeks [7]. To further define the onset of the metabolic regulation, we analyzed FAO genes and 1C genes at 10 weeks (no cardiac phenotype) and at 30 weeks (established cardiomyopathy) of age. A significant downregulation of *Cpt2* and simultaneous upregulation of *Mthfd2* occurred at both time points (Supplementary Fig. 5G and H). We also tested CPT2 protein levels in 10- and 30-week-old mice and found that CPT2 is already significantly downregulated at an early time point of 10 weeks (Supplementary Fig. 5I and  J). These data indicate that the regulation of both pathways are regulated before the onset of a cardiac phenotype, which occurs after 10 weeks of age [7]. To substantiate the metabolic remodeling in the TAZ10 iPSC-CM model, we confirmed higher gene expression and protein levels of *MTHFD2* (Fig. 3I, J). MTHFD2 was also upregulated and in a myocardial sample from one BTHS patient compared with one healthy donor and with two patients with ischemic- or idiopathic dilated cardiomyopathy, respectively (Fig. 3K, L, Supplementary Fig. 5 K).

**Fig. 4:**
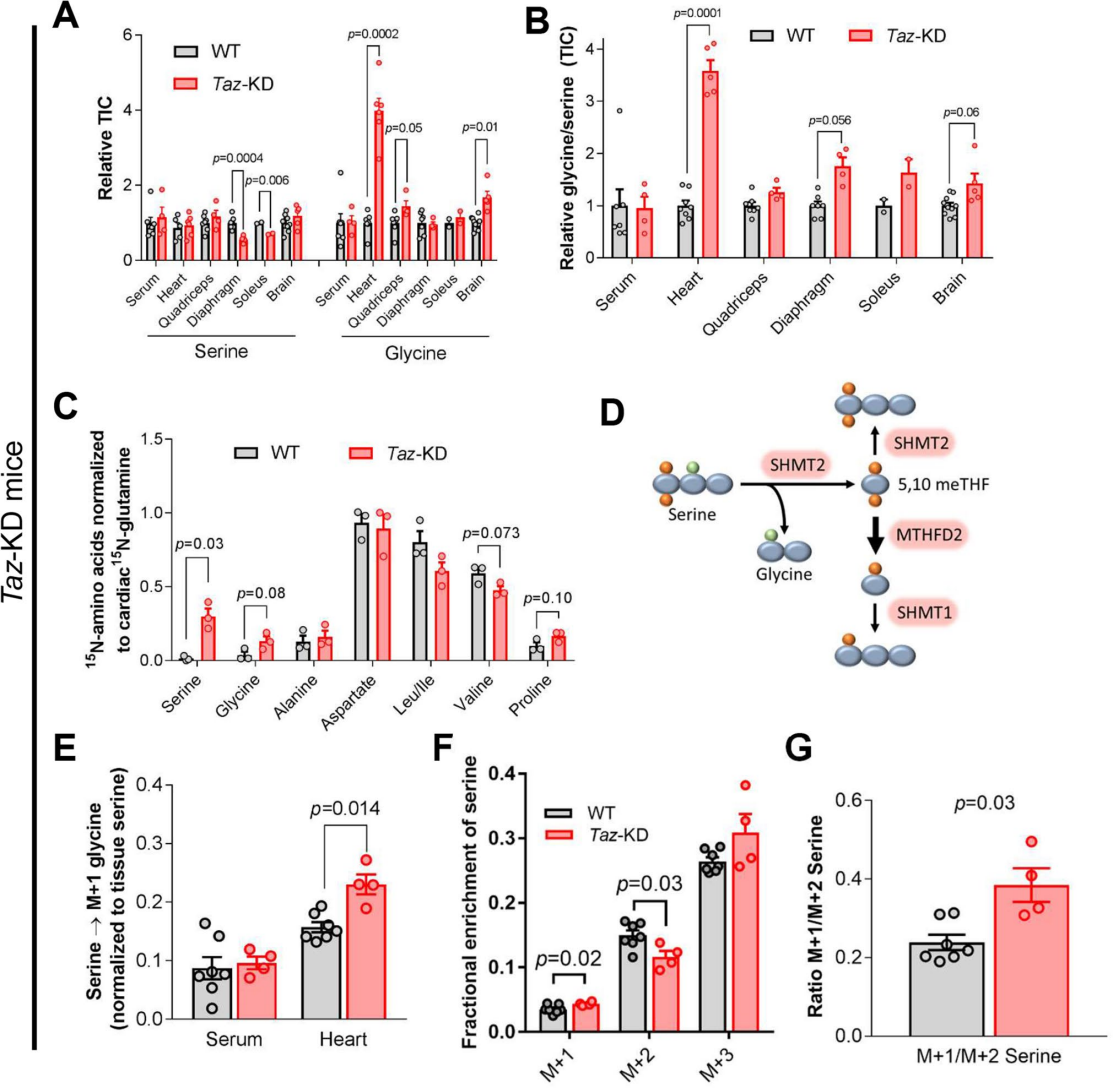
Serine and 1C metabolism are activated in *Taz*-KD hearts.** A** Mass spectrometry analysis of serine and glycine levels in different tissues of 10-week-old *Taz*-KD mice normalized to WT mice. *n* = 3–7 for WT and *n* = 3–4 for *Taz-*KD. **B** Glycine/serine ratios calculated from measurements in A (TIC, total ion chromatogram).** C** Cardiac amino acid labeling from [U-^13^C,^15^N]glutamine infusion. Metabolite enrichment was normalized to the enrichment of cardiac [^15^N]glutamine. *n* = 3 per genotype.** D** Scheme of [2,3,3-^2^H]serine metabolic pathways. **E** Heart and serum glycine labeling from [2,3,3-^2^H]serine infusion. Metabolite enrichment was normalized to the enrichment of tissue [2,3,3-^2^H]serine. *n* = 7 for WT and *n* = 4 for *Taz-*KD. **F** Cardiac serine labeling from [2,3,3-^2^H]serine infusion. *n* = 7 for WT and *n* = 4 for *Taz-*KD. **G** Ratio of M + 1/M + 2 labelled serine calculated from experiment in (**F)**. *n* = 7 for WT and *n* = 4 for *Taz-*KD. Data are mean ± SEM; *n*-numbers are numbers of hearts or animals; statistical significance was determined with two-tailed Student’s t-test

We subsequently sought to interrogate whether transcriptional changes result in increased flux through the serine biosynthetic pathway and 1C metabolism in vivo. Mass spectrometry revealed a substantial shift of the glycine/serine ratio towards increased glycine levels in different tissues, particularly in the heart (Fig. 4A, B), suggesting an increased glycine uptake and/or supplementation of serine by the serine pathway and its subsequent conversion to glycine. To clarify increased serine pathway activity in vivo, we analyzed metabolic fluxes via [U-^13^C,^15^N]glutamine, or [2,3,3-^2^H]serine infusion through the jugular vein in *Taz*-KD and WT mice [45, 90]. Infusion of labeled [U-^13^C,^15^N]glutamine revealed an increased transfer of the labeled amino group onto phosphohydroxypyruvate via the upregulated enzyme PSAT1 to form serine in *Taz*-KD hearts, also in line with an increased flux through the serine pathway (Figs. 3B, 4C). Upon jugular vein infusion, [2,3,3-^2^H]-labeled serine is converted into glycine by the mitochondrial serine hydroxymethyl transferase (*Shmt2*, log2(FC) = 1.37; *p* < 0.0001, Fig. 3B), and determination of M + 1 ^2^H-labeled glycine allows quantitative analyses of SHMT2-mediated conversion of serine to glycine (Fig. 4D). Increased M + 1 labeled glycine indicated significantly increased SHMT2 activity in *Taz*-KD hearts (Fig. 4E). Finally, MTHFD2 mediates the oxidation of 1C metabolites and the consequent loss of one deuterium. Oxidized 1C units compete with reduced forms for reversion to serine. Quantification of fractional enrichment of M + 1 to M + 2 serine in the *Taz*-KD mouse heart demonstrated a marked increase in myocardial MTHFD2 activity (Fig. 4F, G). In summary, these results demonstrate the increased activity of the de novo serine biosynthetic pathway and the mitochondrial arm of 1C metabolism in the hearts of *Taz*-KD mice in vivo.

### The ISR is activated in heart and skeletal muscle of *Taz*-KD mice

**Fig. 5:**
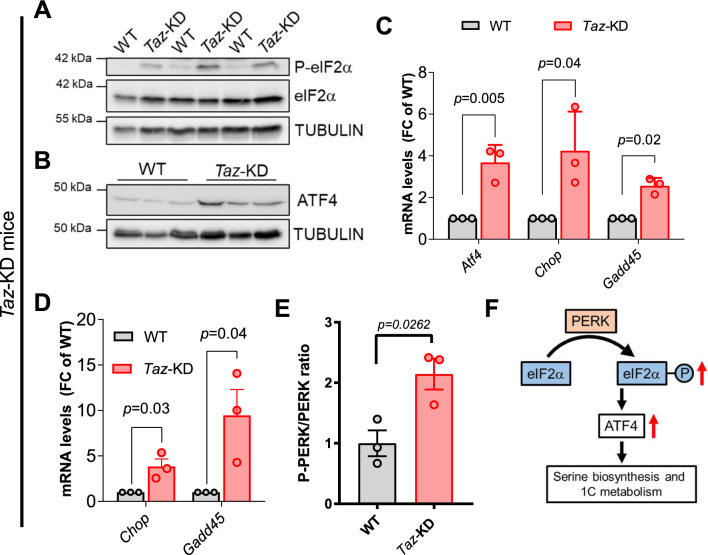
The ISR is activated in heart of *Taz*-KD mice. **A** Western blot analysis of phosphorylated and total eIF2α and TUBULIN as loading control in cardiac lysates. *n* = 3 per genotype. **B** Western blot analysis in cardiac lysates. *n* = 3 per genotype. **C** Cardiac mRNA levels of indicated genes normalized to *Gapdh.* *n* = 3 per genotype. **D** mRNA levels normalized to *Gapdh* in cardiac myocytes. *n* = 3 per genotype. **E** Quantification of western blot analysis of phosphorylated and total PERK protein in cardiac lysates. *n* = 3. **F** Scheme of the ISR signaling pathway. Data are mean ± SEM; *n*-numbers are numbers of hearts or animals; statistical significance was determined with unpaired Student’s *t*-test

Many of the genes upregulated in the cardiac transcriptome of *Taz*-KD mice are under transcriptional control of the basic leucine zipper transcription factor ATF4 (log2(FC) = 2.16; *p* < 0.0001), the key transcriptional effector of the retrograde ISR pathway [80]. Activation of the ISR is triggered by different sensor kinases that converge onto eIF2α phosphorylation, which in turn initiates ATF4 signaling. We detected increased eIF2α phosphorylation as well as increased *Atf4* mRNA and protein levels in the heart of *Taz*-KD mice, confirming activation of the ISR (Fig. 5A–C, Supplementary Fig. 6A, B). Furthermore, qPCR analyses revealed upregulation of the canonical ATF4 target genes *Chop* (log2(FC) = 1.95; *p* < 0.0001) and *Gadd45* (log2(FC) = 1.35; *p* < 0.0001; Fig. 5C) in *Taz*-KD hearts. ATF4 may induce its own transcription, particularly related to a positive feedback loop involving the ATF4 target Nuclear Protein 1 (NUPR1) [46]. To determine whether ATF4 activation indeed occurs in cardiac myocytes, we isolated adult ventricular myocytes from *Taz*-KD and WT mouse hearts. Gene expression analysis confirmed upregulation of *Chop* and *Gadd45* specifically in *Taz-*KD myocytes (Fig. 5D). Subsequently, we sought to identify the kinase responsible for eIF2α phosphorylation. Among the different kinases that phosphorylate eIF2α, we detected increased levels of phosphorylated PKR-like endoplasmic reticulum kinase (PERK) in *Taz*-KD mouse hearts (Fig. 5E, Supplementary Fig. 6C). PERK is an ER membrane-integrated kinase and phosphorylates eIF2α at Ser51 in response to ER stress [41]. Overall, these results demonstrate activation of the eIF2α/ATF4 signaling axis in *Taz*-deficient cardiac myocytes and pinpoint the ER stress sensor PERK as one potential driver of ISR activation (Fig. 5F).

We then sought to assess whether transcriptional changes similar to those observed in the heart also occur in skeletal muscle of *Taz*-KD mice. We identified 2604 differentially regulated genes by RNA sequencing (Supplementary Fig. 6D). A comparison with the cardiac transcriptome revealed a substantial overlap of transcriptional changes occurring in skeletal muscle, with 912 differentially regulated genes in both tissues compared with WT (Supplementary Fig. 6E, F). Akin to the heart, genes encoding enzymes of serine biosynthesis and 1C metabolism were upregulated in skeletal muscle of *Taz*-KD vs. WT mice (Supplementary Fig. 6F). This included upregulation of *Mthfd2*, *Psat1*, and the ATF4 target gene *Gadd45*, which we confirmed by qPCR (Supplementary Fig. 6G), and elevated protein levels of MTHFD2 (Supplementary Fig. 6H).

### ATF4 induces upregulation of 1C metabolism genes in *Taz*-deficient MEFs and iPSC-CM

**Fig. 6:**
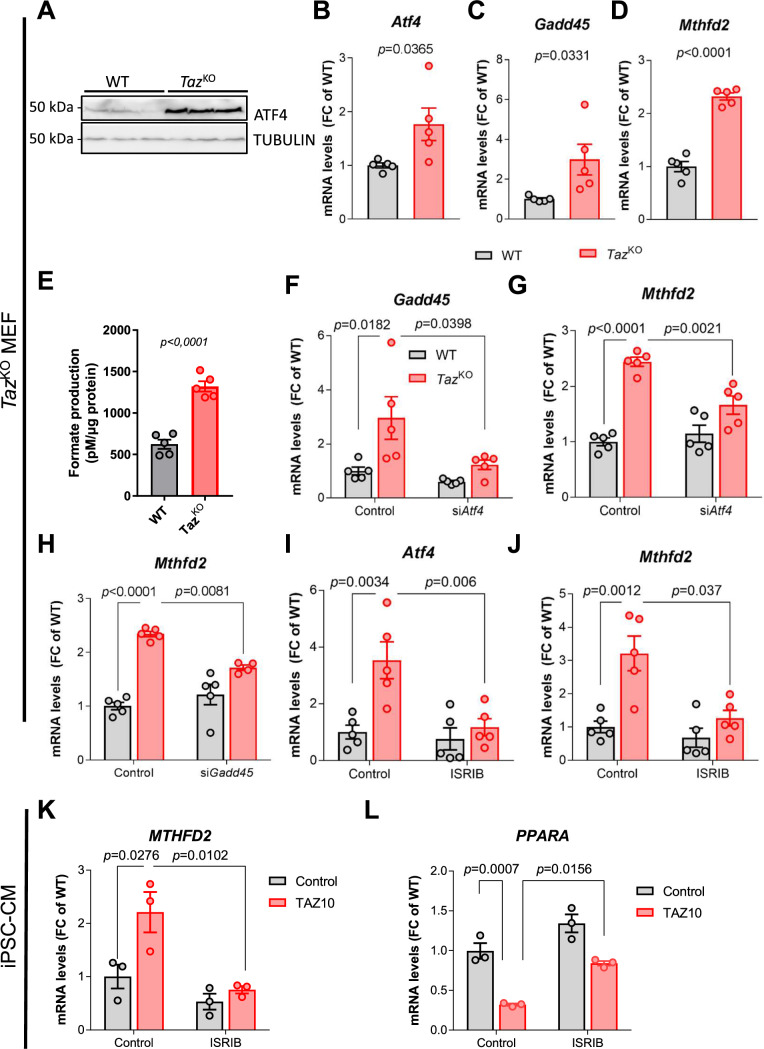
ATF4 induces upregulation of 1C metabolism genes in tafazzin-deficient MEFs and iPSC-CM. **A** Western blot analysis of ATF4 and TUBULIN in WT and *Taz*^KO^ MEFs. **B-D** mRNA levels of indicated genes in MEFs normalized to mS12. *n* = 5 per genotype.** E** Formate levels in the supernatant of MEF culture, normalized to total protein levels as a measure of cell density, *n* = 3. **F, G** mRNA levels of indicated genes in MEFs treated with siRNA against *Atf4* or control normalized to mS12. *n* = 5. **H** mRNA levels of *Mthfd2* in MEFs treated with siRNA against *Gadd45* or control normalized to mS12. *n* > / = 4.** I**, **J** mRNA levels of indicated genes in MEFs treated with ISRIB or DMSO normalized to mS12. *n* = 5. **K, L** mRNA levels of indicated genes in iPSC-derived CM treated with ISRIB or DMSO normalized to L28. *n* = 3. Data are mean ± SEM; *n*-numbers are numbers of independent experiments for panels **B**–**J** and technical replicates in panels K and L; statistical significance was determined with unpaired Student’s *t*-test in panels B to E, and with one-way ANOVA followed by Tukey’s multiple comparison for panels F to L

To interrogate whether activation of ATF4 signaling is a general response to *Taz* deficiency, we assessed ISR signaling in a *Taz-*deficient mouse embryonic fibroblast (MEF) cell line obtained by CRISPR/Cas9-mediated gene deletion of *Taz* (*Taz*^KO^) [18]. ATF4 protein levels were clearly increased in *Taz*^KO^ vs. untreated WT MEFs (Fig. 6A, Supplementary Fig. 7A), but remained lower compared to the ER stress inducer tunicamycin (Supplementary Fig. 7B, C). Accordingly, gene expression of *Atf4* and its target genes *Gadd45* and *Mthfd2* was strongly upregulated in *Taz*^KO^ MEFs (Fig. 6B–D). The serine biosynthesis enzyme PSAT1 is upregulated on the protein level (Supplementary Fig. 7D). In mammalian cells, the mitochondrial arm of 1C metabolism produces formate from serine, while the cytosolic arm uses formate for nucleotide synthesis [11, 68]. In support for increased 1C metabolism, formate levels were increased in *Taz*^KO^ vs. WT MEFs (Fig. 6E).

To confirm that ATF4 signaling dictates upregulation of 1C metabolism genes, we silenced *Atf4* by small interfering RNA (siRNA). siRNA-mediated knockdown of *Atf4* abrogated the upregulation of *Gadd45* and *Mthfd2* in *Taz*^KO^ MEFs (Fig. 6F, G Supplementary Fig. 7E). Also silencing the ATF4-dependent transcription factor *Gadd45* abolished *Mthfd2* upregulation, indicating that the latter is under control of the ATF4/GADD45 signaling axis (Fig. 6H, Supplementary Fig. 7F). To further substantiate the involvement of the ISR in the transcriptional changes observed in *Taz*-deficient MEFs, we blocked the ISR with ISRIB, a small molecule inhibitor of phospho-eIF2α activity [72]. In fact, ISRIB reverted *Atf4* and *Mthfd2* expression in *Taz*^KO^ MEFs to WT levels (Fig. 6I, J). Upregulation of the key 1C metabolism gene *MTHFD2* was also evident in TAZ10 iPSC-CM, and treatment with ISRIB blunted its upregulation (Fig. 6K). Since PGC-1α expression is regulated by ATF4 in adipose tissue, we used the iPSC-CM model to analyze the cross-talk between ISR activation and the downregulation of FA oxidation enzymes [83, 84]. ISRIB treatment increased *Ppara* gene expression, indicating that the ISR induces transcription of 1C metabolism genes while repressing genes involved in FA oxidation in human cardiac myocytes (Fig. 6L).

To better characterize the extent of metabolic changes in the MEF *Taz*^KO^ model, we analyzed the gene regulation of glucose transporters. *Glut4*, but not *Glut1*, is significantly increased in *Taz*^KO^ MEFs, but blocking ISR had no effect on glucose transporter expressions (Supplementary Fig. 7G). Furthermore, no change of O-linked N-actetylglucosamine (O-GlcNAc) was observed in *Taz*^KO^ MEFs compared to WT MEFs (Supplementary Fig. 7H). Autophagy activation, as determined by LC3-II/LC3-I ratio, is decreased in *Taz*^KO^ MEFs and does not further increase upon lysosomal inhibition with bafilomycin, confirming a defect in the activation of autophagy (Supplementary Fig. 7I). ISRIB application does not change the LC3-II/LC3-I ratio, indicating that the ISR activation is not causative to changes in autophagy (Supplementary Fig. 7 J). Finally, ISR activation does not predispose to glucose deprivation-induced cell death (Supplementary Fig. 7 K).

### Glutamate metabolism is rewired in *Taz*-KD hearts

**Fig. 7:**
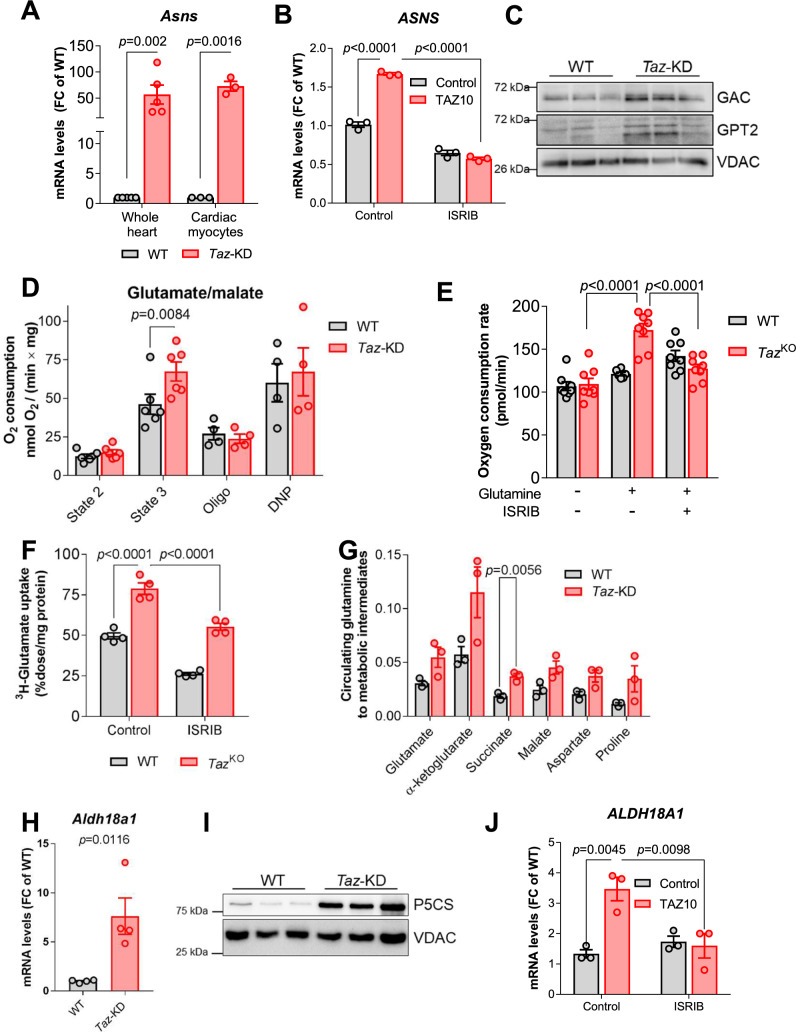
Rewiring of glutamate metabolism in *Taz*-KD hearts. **A** mRNA levels in whole hearts (*n* = 5) or isolated mouse cardiac myocytes (*n* = 3), normalized to *Gapdh*. **B** mRNA levels in iPSC-derived CM treated with ISRIB or DMSO, normalized to *ACTB*, *n* = 3. **C** Western blot analysis of cardiac mouse mitochondria. **D** OCR of isolated cardiac mitochondria from 20-week-old WT and *Taz*-KD mice supplied with glutamate and malate in the absence (state 2) and presence (state 3) of ADP (1 mM). Subsequent measurements are done after oligomycin and DNP administration. *n* = 6 per genotype. **E** OCR of MEFs with or without glutamine and the administration of ISRIB. *n* > / = 6. **F** Uptake of ^3^H-glutamate in WT and *Taz*^KO^ MEFs treated or not with ISRIB. *n* = 4. **G** Cardiac metabolite levels labeled from [U-^13^C, ^15^N]glutamine infusion normalized to the enrichment of tissue [U-^13^C,^15^N]glutamine. *n* = 3. **H** Cardiac mRNA levels of *Aldh18a1* in WT and *Taz*-KD mice normalized to *Gapdh*. *n* = 5. **I** Western blot analysis in cardiac lysates. *n* = 3 per genotype. **J** mRNA levels in iPSC-derived CM treated with ISRIB or DMSO as a control normalized to L28. *n* = 3. Data represent mean ± SEM; *n*-numbers are numbers of animals for panels A, C, D, G, H and I numbers of independent replicates for panels E and F and technical replicates for B and J. Statistical significance was determined with unpaired Student’s t-test in panels A and H, with two-way ANOVA followed by Bonferroni’s multiple comparisons test for panel D and with one-way ANOVA followed by Tukey’s multiple comparisons test for panels B, E, F, and K

In agreement with the pivotal role of ISR in amino acid metabolism, analysis of the *Taz*-KD cardiac transcriptome indicates upregulation of many amino acid transporters, in particular the glutamine and glutamate transporters *Asct1* and *Slc38a1* and the glycine transporter *Slc6a9* [40, 42]. We confirmed upregulation of these amino acid transporters in *Taz*-KD hearts (Supplementary Fig. 8A). Glutamine is known to play a particular role in cancer cells, where the increased abundance of this amino acid is exploited for anaplerotic replenishing of the Krebs cycle through the conversion of glutamate to α-ketoglutarate (Fig. 1) [52]. Interestingly, one of the most strongly upregulated genes in the transcriptome is the asparagine synthase ASNS (*Asns*, log2(FC) = 5.76; *p* < 0.0001, Fig. 3A). This enzyme converts aspartate into asparagine to allow the deamination of glutamine to glutamate for its further conversion in metabolism. Other transaminases, such as BCATm were not upregulated (*Bcat2*, log2(FC) = 0.23; *p* < 0.0101). Elevated activity of ASNS may explain increased asparagine levels in BTHS patients [82]. Increased levels of asparagine synthase was confirmed by qPCR in mouse heart, isolated cardiac myocytes and iPSC-CM (Fig. 7A, B). ISRIB treatment of TAZ10 iPSC-CM confirmed the ISR as a primary regulator of *ASNS* expression (Fig. 7B). We also detected increased protein levels of the glutaminase splice variant glutaminase C (GAC) and increased mRNA as well as protein levels of the glutamic-pyruvic transaminase 2 (*Gpt2,* log2(FC) = 0.81; *p* < 0.0001; Figs. 1, 7C and Supplementary Fig. 8B), which catalyze the two sequential reactions converting glutamine into glutamate and subsequently into the Krebs cycle intermediate α-ketoglutarate [28].

To assess the functional relevance of these changes, we determined glutamate/malate-supported respiration in isolated cardiac mitochondria and observed a robust increase in maximal ADP-stimulated respiration in *Taz*-KD vs. WT mitochondria, confirming the increased capacity for glutamate oxidation of *Taz*-KD hearts (Fig. 7D). Moreover, cellular respiration of *Taz*^*K*O^ MEFs was increased by glutamine supplementation in the cell culture medium, which did not affect respiration of WT MEFs (Fig. 7E). The glutamine transaminase inhibitor aminooxyacetate (AOA) had a more pronounced suppressive effect on O_2_ consumption rate of *Taz*^KO^ compared to WT MEFs (Supplementary Fig. 8C, D). Moreover, in order to test, if preferred glutamine metabolism is dependent on ISR signaling in MEFs, we treated these with the specific PERK kinase (PERKi) inhibitor GSK2606414. Normalized to non-treated controls, only *Taz*^KO^ MEFs, but not WT MEFs showed a significant decrease upon PERKi treatment, indicating that ISR activation regulates glutamine metabolism in CL-deficient cells (Supplementary Fig. 8E, F). To assess whether increased glutamate uptake and utilization is also driven by ATF4 signaling, we analyzed ^3^H-glutamate uptake in MEFs and observed that this was increased in *Taz*^KO^ vs. WT MEFs and reduced by ISR inhibition with ISRIB (Fig. 7F). To interrogate whether glutamine flux into the Krebs cycle is increased also in vivo, we subjected *Taz*-KD and WT mice to infusion of labeled [U-^15^N,^13^C]glutamine and detected increased incorporation of glutamine carbon into the Krebs cycle intermediates downstream of α-ketoglutarate (Fig. 7G). Glutamate, converted into oxaloacetate via the Krebs cycle, also supports the serine biosynthetic pathway via conversion of oxaloacetate to phosphoenolpyruvate mediated by phosphoenolpyruvate carboxykinase (PCK2). *Pck2* is upregulated in *Taz*-KD hearts (Supplementary Fig. 8G) (*Pck2*, log2(FC) = 1.96; *p* < 0.0001), which is typical for heart disease [61].

Supporting a role of glutamate in the intermediate metabolism, we also followed its conversion into proline via two reactions catalyzed by delta-1-pyrroline-5-carboxylate synthetase (P5CS, gene *Aldh18a1*) and the pyrroline-5-carboxylate reductase (PYCR1, Fig. 1). *Aldh18a1* (log2(FC) = 3.28; *p* < 0.0001) and *Pycr1* (log2(FC) = 3.30; *p* < 0.0001) were strongly upregulated in *Taz*-KD hearts according to RNA sequencing (Fig. 3A). Accordingly, we found increased *Pycr1 and Aldh18a1* mRNA and P5CS protein levels in the heart of *Taz*-KD mice (Fig. 7H, I and Supplementary Fig. 8H, I). *ALDH18A1* was upregulated in patient-derived iPSC-CM and ISRIB treatment revealed its dependency on the ISR signaling pathway (Fig. 7J). Also in vivo, we detected a trend toward increased incorporation of labeled [U-^15^N,^13^C]glutamine carbon into proline in *Taz*-KD hearts (Fig. 7G).

### ATF4 induces cysteine uptake via the xCT system to support glutathione biosynthesis

**Fig. 8:**
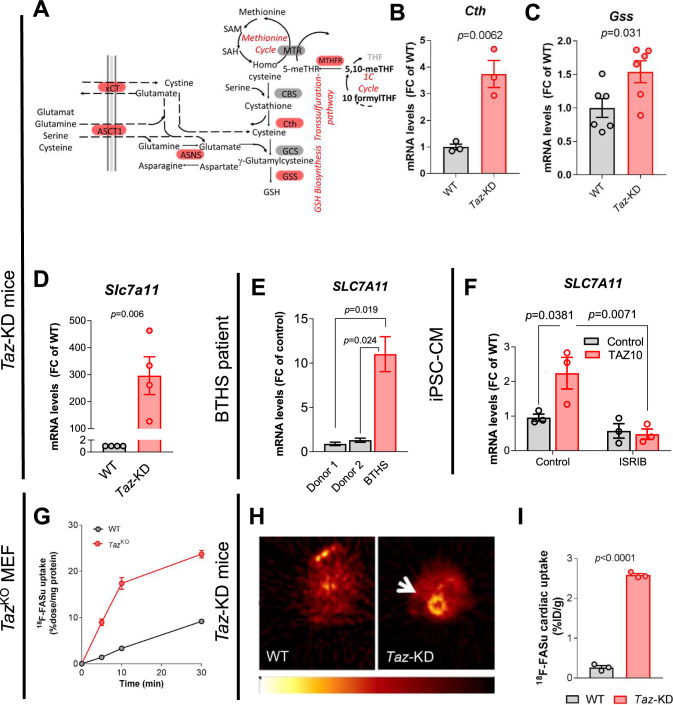
ATF4 induces cysteine uptake via the xCT system to support glutathione biosynthesis. **A** Cartoon of metabolic pathways. Significantly upregulated genes in *Taz*-KD vs. WT hearts highlighted in red. **B, C** Cardiac mRNA levels in mice normalized to *Gapdh.* *n* = 3 per genotype for B and *n* = 6 per genotype for C. **D** Cardiac mRNA levels in mice normalized to *Gapdh.* *n* = 4. **E** mRNA levels normalized to L28 in myocardial samples from one BTHS patient and 2 healthy controls. Technical replicate, *n* = 3.** F** mRNA levels of in iPSC-derived CM from one BTHS patient (TAZ10) or control normalized to *ACTB*, treated with ISRIB or DMSO. *n* = 3. **G** Uptake of the xCT system-specific radiotracer ^18^F-FASu into MEFs, measured by scintillation counting. **H** Representative PET-CT image of cardiac ^18^F-FASu uptake in 34-week-old mice.** I** Quantification of cardiac ^18^F-FASu uptake in mice. Data are mean ± SEM; n-numbers are numbers of animals for panels B–D and I, and numbers of technical replicates for panels F. Statistical significance was determined with unpaired Student’s *t*-test in panels B–D and I and with one-way ANOVA followed by Tukey’s multiple comparison for panels E and F

ATF4-driven activation of serine, 1C and glutamate metabolism serves numerous functions, including the production of reducing equivalents and the biosynthesis of glutathione (GSH) for H_2_O_2_ elimination [64]. Elevated levels of the antioxidant GSH, GSSG and the precursors of its biosynthesis pathway cystathione and γ-glutamylcysteine were measured in *Taz*^KO^ MEFs (Supplementary Fig. 9A). GSH is a tripeptide composed of glutamate, cysteine, and glycine. The central amino acid cysteine can be provided by the transsulfuration of homocysteine and serine via cystathione and the subsequent conversion to cysteine via the enzyme cystathione lyase (*Cth*, Fig. 1), which is upregulated on gene expression level (Fig. 8A, B). Cycteine and glutamate then form γ-glutamylcysteine, which is then converted to glutathione via the glutathione synthase (*Gss*), which is also upregulated in *Taz*-KD mouse hearts (Fig. 8C). Cysteine is the rate-limiting precursor for GSH synthesis and is mostly imported as its oxidized form (cysteine) via a cystine/glutamate antiporter system xCT, located in the cell membrane (Fig. 1). Increased glutamate drives system xCT cystine/glutamate antiporter activity in cancer cells [52], where its transcription is controlled by ATF4 [17]. We observed that the gene encoding system xCT *(Slc7a11)* was markedly upregulated in *Taz*-KD hearts (log2(FC) = 5.27; *p* < 0.0001) and skeletal muscle according to RNA sequencing, which we confirmed by qPCR (Fig. 8D, Supplementary Fig. 9B). We also detected *SLC7A11* upregulation in the myocardium of one BTHS patient (Fig. 8E) as well as in TAZ10 iPSC-CM and *Taz*^KO^ MEFs, which could be reverted by ISR inhibition with ISRIB in iPSC-CM and with siRNA-mediated knockdown of *Atf4* in *Taz*^KO^ MEFs (Fig. 8F, Supplementary Fig. 9C, D).

To assess whether *Slc7a11* upregulation results in increased activity of the system xCT, we measured uptake of the xCT-specific radiotracer ^18^F-FASu in vitro in MEFs and in vivo in *Taz*-KD mice*.* Compared to WT, uptake of ^18^F-FASu was substantially increased in *Taz*^KO^ MEFs (Fig. 8G) and in particular, in hearts of *Taz*-KD mice in vivo (Fig. 8H, I). Increased cardiac and skeletal muscle cysteine uptake may contribute to decreased circulating cystine (the oxidized form of cysteine) in BTHS patients [82]. Glutathione derived from internalized cysteine serves to reduce H_2_O_2_ to H_2_O, a reaction catalyzed by glutathione peroxidase (GPX). Together with the previously reported elevated levels of GPX1, also GPX4 protein levels are elevated in hearts of *Taz*-KD mice (Supplementary Fig. 9E) [6]. *Gpx1* upregulation was also detected in *Taz*^KO^ MEFs and controlled by ISR signaling (Supplementary Fig. 9F).

Together, these data suggest that in *Taz*-deficient hearts, displaying a similar heart failure phenotype as in stable patients with BTHS [20], activation of the ISR increases glutamate uptake that serves (1) to fuel the Krebs cycle through anaplerosis, (2) to produce other amino acids such as asparagine and proline, and (3) as a driving force to facilitate the uptake of cysteine via the system xCT, which is utilized for the production of glutathione (Fig. 1). These processes in concert with ISR-dependent upregulation of 1C metabolism may compensate for the primary cardiac mitochondrial defects in BTHS, where CL deficiency compromises the uptake of the primary fuel (i.e., fatty acids; this study) and the physiological activation of Krebs cycle dehydrogenases during workload transitions through mitochondrial Ca^2+^ uptake [6].

## Discussion

Mitochondrial dysfunction in BTHS causes cardiomyopathy and muscle fatigue, which limits exercise capacity and poses a high risk for sudden cardiac death [32, 74]. Here, we identify how alterations in cardiac substrate metabolism to some extent compensate for these mitochondrial defects (Fig. 1). First, we reveal reduced FA uptake and β-oxidation in hearts of *Taz*-deficient mice in vivo and trace this to downregulation of CPT2 and ACADVL. Second, we report increased glucose uptake into *Taz*-KD hearts in vivo, with increased funneling of glycolytic intermediates into the serine biosynthetic pathway and 1C metabolism. Third, we discover a strong concerted upregulation of cardiac glutamate transporters and the system xCT, which exchanges glutamate for cysteine towards glutathione synthesis, whereas glutamate is also channeled into anaplerotic pathways towards ATP production in mitochondria. And fourth, this well-concerted metabolic rewiring is coordinated by ATF4.

### Impaired cardiac fatty acid uptake and oxidation and metabolic remodeling

Fatty acids (FA) are the major source for ATP in the human heart [62]. In patients with BTHS, reduced myocardial FA extraction at rest and exercise-induced FA oxidation rates correlate with lower PCr/ATP ratios and cardiac dysfunction [12, 13]. In contrast, a recent study revealed increased myocardial FA oxidation rates in isolated ex vivo hearts from *Taz*-KD mice [38]. To resolve this contradiction, we used the novel radiotracer ^18^F-FTOa in vivo and observed substantially reduced FA oxidation*.* We identified reduced protein levels and lower gene expression of CPT2 as potential limiting factors for cardiac FA transport and oxidation in the *Taz*-KD mouse model. Lower protein levels of CPT2 result either from transcriptional regulation, or from a defect in the integration of proteins into the inner mitochondrial membrane as a consequence of changes in the lipid bilayer. Reduced levels of CPT2 correspond with reduced palmitoylcarnitine-driven respiration, confirming other studies in *Taz*-KD mice [49, 50, 54, 67] and a recently developed mouse model carrying the patient TAZ^G197^ mutation [19]. Accumulation of long-chain acylcarnitines as a result of a FAO block was found in patient-derived iPSC-CM, carrying the TAZ517delG mutation [31]. Reduced fatty acid oxidation was recently linked to reduced levels of coenzyme A (CoA) due to reduced levels of acyl-CoA synthase. We are able to support this finding in the transcriptomic data set (log2(FC) =  – 0,54; *p* = 1,37E-07) and by qPCR (Supplementary Fig. 1 J) [54]. Interestingly, CoA deficiency might contribute to reduced fatty acid oxidation, while the involvement of CoA in other metabolic processes, including glutamate metabolism, remains unaltered in BTHS [19, 31]. Since deficiencies of either ACADVL [8, 77] or CPT2 [59] can cause cardiomyopathy and arrhythmias in newborns and early infancy, the substantial reduction of FA uptake and oxidation in BTHS hearts likely contributes to the cardiomyopathy phenotype. However, it needs to be discussed that respiratory defects are not constrained to fatty acids in Barth syndrome, as evidence for reduced succinate and ADP driven respiration has been provided by multiple studies in all age groups [27, 47, 71]. Succinate-driven respiration was ameliorated by administration of SS-31 (elamipretide), a mitochondria-targeted tetrapeptide, tested in clinical trials, which was shown not only to interact with CL but also to metabolic enzymes in the Krebs cycle [16, 78, 79]. Pyruvate/malate-driven respiration was reduced in 7–8-month- [47] and in 4–5-month-old mice [54], whereas no difference was found in 2-month-old mice [49]. Mitochondrial fractionation revealed that pyruvate/malate driven respiration was particularly reduced in subsarcolemmal (SSM), but not in interfibrillar mitochondria (IFM) [50]. Reduced levels of succinate-, pyruvate- and palmitate-driven respiration were also confirmed in mice carrying cardiac myocyte-specific *Taz* gene ablation [93]. A compensating upregulation of glutamate metabolism corresponds with observations in studies analyzing isolated mitochondria from *Taz*-KD hearts [49, 54] and iPSC-CM [31], whereas other studies observed only moderate changes in glutamate metabolism [50].

Metabolic alterations are associated with increased H_2_O_2_ emission and increased oxidative damage in some studies [54, 85], in contrast to other studies [35] and our previous experience [7]. We addressed the hypothesis that the increase in myocardial glucose uptake observed in BTHS patients [13] and in the *Taz*-KD mouse model compensated for impaired FA oxidation. While glucose uptake was clearly upregulated in *Taz*-KD mouse heart (Fig. 2C), regulation of glucose transporter followed a more complex pattern (Supplementary Figs. 1E and 7G). Increased glucose uptake bears the risk of rerouting glucose also into the hexosamine pathway, which would result in an increased level of O-GlcNAcylation of proteins, which has been associated with heart failure before [81]. However, we did not detect any increase in O-GlcNAcylation in *Taz*-KD hearts (Supplementary Fig. 1 K) or in *Taz*^KO^ MEFs (Supplementary Fig. 7H). In agreement with this, expression of the GlcNAc transferase OGT was almost unchanged in the mouse cardiac transcriptome (log2(FC) = 0,18; *p* = 0,0279).

The activity of the PDH complex is reduced in BTHS, as found in *Taz*-KO C2C12 myoblast cell models [38, 56] and in *Taz*-KD hearts ([38] and own data not shown). Activity of the PDH complex is inhibited by phosphorylation, which is relieved by the Ca^2+^-activated PDH phosphatase. We speculate that defective mitochondrial Ca^2+^ uptake contributes to the decrease in PDH activity and aggravates the redox and bioenergetic deficit [7, 53]. This regulatory mechanism is lost when mitochondria are removed from their intracellular context, explaining the unchanged respiration of *Taz*-KD cardiac mitochondria on pyruvate/malate (Fig. 2D).

### CL deficiency activates the integrated stress response (ISR)

Several stressors trigger ISR activation in the heart, including ischemia/reperfusion injury [91], pressure overload [88], and defects in FA oxidation [69]. A role for the activation of the stress sensor kinase HRI in context of mitochondrial dysfunction was discussed recently, however, our data rather argue for a role of PERK in BTHS [33, 39, 94]. The mechanistic link between cardiac stressors and ISR activation remains poorly understood [88, 91]. Oxidative stress is a potent inducer of the ISR and might explain its activation in the context of cardiac pressure overload. There is a considerable number of studies that observed increased propensity to produce ROS in different experimental models of BTHS [37, 47, 54, 85]. However, we [6] and others [36] did not observe an increase in mitochondrial ROS formation in *Taz*-KD vs. WT hearts in vitro or in vivo. While some studies found increased ROS levels in *Taz*-deficient iPSC-CMs [85] and *Taz* knock out* (Taz-*KO) hearts [58], our data agree with a recent study that observed no increase in ROS formation at 11 different sites within cardiac and skeletal muscle mitochondria of *Taz*-KD mice [35]. In mouse models of defective FA oxidation, the inciting event might be a shortage of amino acids, which is a known inducer of the ER-resident stress-induced kinase GCN2 [69]. However, direct evidence of GCN2 activation in the latter models is lacking, and we could not detect GCN2 phosphorylation in mouse hearts with commercially available antibodies.

The extensive metabolic reprogramming induced by ATF4 reroutes the glycolytic intermediates toward serine synthesis and 1C metabolism, but no evidence was found that ATF4 regulates glucose uptake (Fig. 1). Buttressing our results in the *Taz*-KD mouse model, elevated PHGDH protein levels were observed in myocardial tissue from BTHS patients [15]. Our experiments in the iPSC-CM and *Taz*^KO^ MEF models of BTHS identify the eIF2α/ATF4 branch of the ISR as the driver of these transcriptional changes, which could be completely reverted by ISR inhibition with ISRIB or ATF4 silencing. Furthermore, we confirmed that flux through these pathways is enhanced in vivo in the *Taz*-KD model of BTHS. In addition, although *Ppara* is not a known target gene for ATF4, the observation that *Ppara* gene expression is partly rescued upon ISR inhibition with ISRIB in the iPSC-CM model of BTHS suggests that ATF4 signaling also to a certain extent contributes to transcriptional suppression of FA oxidation (Fig. 6L).

Experimental evidence indicates that the primary function of ATF4-induced serine biosynthesis and 1C metabolism is to support glutathione biosynthesis and the production of reducing equivalents for ROS elimination, conferring additional protection to the heart against oxidative stress [65, 88]. Interestingly, a particular bias of the cytosolic (SHMT1) vs. mitochondrial (SHMT2) isoform of the serine to glycine converting enzymes indicates that serine is preferentially fluxed into mitochondria (Fig. 3E, G). Increased PHGDH and SHMT2 proteins and decreased serine and glycine levels were detected in myocardial tissue from patients with end-stage heart failure who responded to left ventricular assist device (LVAD)-induced mechanical unloading compared with those that did not benefit from LVAD, suggesting that increased flux through these pathways is associated with cardiac recovery [3]. In fact, glutathione has a well-documented protective role in heart failure [63, 89]. Patients with a polymorphism in the glutamate–cysteine ligase have a higher risk for myocardial infarction and dilated cardiomyopathy (DCM) [63]. Mice with a defect in glutathione biosynthesis show exaggerated phenotype to pressure overload-induced hypertrophy, similar to glutathione peroxidase deficient mice [2, 89]. Although glutathione levels were unchanged in *Taz*-KD vs. WT hearts, we previously reported that GPX1 protein levels are twofold higher in *Taz*-KD hearts [6]. Here, we demonstrate that *Gpx1* upregulation is mediated by the ISR in *Taz*^KO^ MEFs (Supplementary Fig. 9F). The absence of oxidative stress in spite of the shortage of reducing equivalents provided by the Krebs cycle [6] could be explained, at least in part, by an increased supply of reduced NADPH from NADP-coupled reactions in 1C metabolism [29]. Altogether, our results suggest that ATF4-induced serine biosynthesis and 1C metabolism may support glutathione biosynthesis and prevent ROS emission from mitochondria.

### The role of glutamate metabolism in Barth syndrome

While cysteine is the rate-limiting substrate for glutathione biosynthesis, the role of system xCT in the heart has not been investigated so far [55]. Glutathione production requires uptake of extracellular cystine (the oxidized form of cysteine) via the cystine/glutamate antiporter system xCT [76]. In a mouse model of cardiac iron overload obtained by cardiac myocyte-specific deletion of the ferritin H gene, cardiac overexpression of Slc7a11 prevented ferroptosis by increasing glutathione levels [30]. Here, we show that activation of the ISR induces *Slc7a11* upregulation in BTHS, and demonstrate that the activity of the system xCT is increased in the *Taz*-KD mouse heart in vivo, revealing a role for this transporter in human cardiomyopathy.

In cancer cells, activation of the system xCT is accompanied by extensive remodeling of glutamate metabolism, because it operates as a cystine/glutamate antiporter. xCT overexpression renders cancer cells highly dependent on glutamine, which is also used for replenishing the Krebs cycle via anaplerosis [52]. Increased anaplerotic flux in *Taz-*KD hearts in vivo and mitochondrial respirometry in vitro indicate a similar rewiring of glutamate metabolism in BTHS. Of note, in patients with BTHS, amino acid turnover is increased [14, 82], and protein supplementation in combination with resistance exercise training improved skeletal muscle strength and quality of life in patients with BTHS [10]. While in particular, plasma glycine and glutamic acid concentrations tended to increase by this protein cocktail, the effects appeared to be related largely to improvement of skeletal muscle function [10]. However, whether the blunted increase of cardiac function during exercise [74] or in response to β-adrenergic stimulation [6] is improved by this treatment is currently unresolved.

In conclusion, we identified activation of the ISR as the key driver of metabolic remodeling in BTHS cardiomyopathy. Transcriptional reprogramming induced by the eIF2α-ATF4 signaling axis activates de novo serine biosynthesis, 1C metabolism and the system xCT to sustain glutathione synthesis and production of reducing equivalents for ROS elimination. This is accompanied by a rewiring of glutamate handling, which is exploited to sustain both cystine uptake for glutathione synthesis and Krebs cycle anaplerosis.

## Methods

### Mice

Maintenance and animal experiments were performed according to the guidelines from the German Animal Welfare Act and approved by the Bayerisches Landesamt für Gesundheit und Lebensmittelsicherheit, Germany (AZ: 55.2.2–2535-804). Doxycycline (625 mg/kg) was administered as part of the standard rodent chow to WT C57BL/6N mice and transgenic (ROSA26H1/tetO-shRNA:TAZ) animals [1, 6, 73]. Doxycycline was withdrawn from female mice for 1 week before mating and during the mating period to avoid male infertility. Doxycycline treatment was resumed upon successful mating (copulatory plugs) and continued until the end of experiment at an age of 30- 50 weeks, unless indicated otherwise. The genotype of the pups was assessed by PCR, as described previously [1]. Mouse line and transgene induction were identical to our previous studies [6]. For analysis of in vivo metabolism, animals were fasted for 12 h. Radiotracers were administered via intraperitoneal (i.p.) injection of 7–15 MBq ^18^F-FDG, or via intravenous (i.v.) injection of 7–15 MBq ^18^F-FTOa, or 7–15 MBq ^18^F-FASu. 5 min prior analysis, anaesthesia was started, using 2% isoflurane. Static 30-min PET imaging (60–90 min) followed by postmortem organ tissue counting was performed using a Inveon PET System, Siemens Medical Solutions (Erlangen, Germany) and Wizard Gamma Counter, PerkinElmer (Waltham, MA).

### Mouse in vivo metabolomics

[U-^13^C] glucose (200 mM in 0.9% NaCl solution, catalog # Cambridge CLM-1396), [2,3,3-^2^H]serine (100 mM in 0.9% NaCl solution, catalog # Cambridge DLM-582) or [U-^13^C, ^15^N]glutamine (100 mM in 0.9% NaCl solution, catalog# Cambridge CNLM-1275) was infused via jugular vein at a rate of 0.1µL/min/g for 2.5 h (glucose and serine infusion) or 4 h (glutamine infusion). Mice were able to move freely in a tethering apparatus (Instech Laboratories) with no access to food during the course of the experiment. To assess venous tracer enrichment, blood was sampled from jugular vein at the end of the experiment. Blood samples were cooled on ice to coagulate and then serum was collected following centrifugation at 16,000 × g for 10 min at 4 °C. Tissues samples were collected by clamping in aluminum foil with a pre-cooled Wollenberger like clamp and immediately transferred into liquid nitrogen. All serum and tissues samples were stored at -80 °C until further analysis.

### Mitochondria isolation from mouse heart tissue

All steps for the mitochondria isolation were performed on ice and the centrifuge was precooled to 4 °C. Frozen heart tissue was cryo-grinded with a ceramic mortar and pistil and transferred to a manual homogenizer. The tissue was homogenized in 2 mL isolation buffer (20 mM Hepes pH7.6, 220 mM mannitol, 70 mM sucrose, 1 mM EDTA) with freshly added 0.5 mM PMSF, complete protease inhibitors (Roche) and phosphatase inhibitors (Roche). Homogenized sample was transferred to a 2 ml tube and centrifuged 15 min at 800 g. The supernatant was transferred to a new 2 ml tube and kept on ice until further usage. The homogenization and centrifugation steps were repeated another two times with the pellet after the centrifugation. After the third homogenization and centrifugation, the pellet was discarded and the collected supernatant was centrifuged at 10,000 g for 10 min. Supernatant was transferred into a new reaction tube and stored at -80 °C until further usage. Mitochondria were washed and resuspended in 100 µL isolation buffer and protein amount was determined using the DC protein assay from BioRad. Mitochondria were used directly for respiration measurements or stored at  – 80 °C. Heart tissue for respiration measurements was homogenized in isotonic isolation solution (225 mM mannitol, 75 mM sucrose, 2 mM HEPES, 1 mM EGTA; pH 7.4) and mitochondria were isolated and washed in suspension solution (225 mM mannitol, 75 mM sucrose, 2 mM HEPES, 1 mM EGTA; pH 7.4). For isolation of mitochondria from skeletal muscle, tissue was homogenized in basic medium (140 mM KCl, 20 mM HEPES, 5 mM MgCl_2_, 1 mM EGTA; pH 7.0) supplemented with 1 mM ATP, 1% BSA and 1 U substilisin A. Mitochondrial isolation from mouse embryonal fibroblasts were performed in THE buffer (300 mM Trehalose, 10 mM KCl, 10 mM HEPES, 1 mM EDTA, 1 mM EDTA, 10 mg/ml BSA) with freshly added 0.5 mM PMSF, complete protease inhibitors (Roche) and phosphatase inhibitors (Roche). Mitochondria pellets were washed and resuspended in THE buffer without BSA.

Oxygen consumption of 400 µg isolated mitochondria was measured in 2 mL volume of standard respiration buffer (137 mM KCl, 2 mM KH_2_PO_4_, 0.5 mM EGTA, 2.5 mM MgCl_2_, 20 mM HEPES, pH 7.2) at 37 °C (Oroboros instruments). State 2 was measured in presence of 5 mM sodium pyruvate and 5 mM sodium malate. Alternatively, 5 mM Malate + 50 µM Palmitoyl-carnitine or 5 mM Malate + 200 µM Octanoyl-carnitine was used for FAO. Respiration (State 3) was stimulated by adding 1 mM ADP. Finally, O_2_ consumption linked to ADP phosphorylation was inhibited by adding the F_1_-F_o_ ATP synthase blocker oligomycin (1.25 µM) and subsequently respiration was uncoupled by titrating DNP in 10 µM steps until complete uncoupling was achieved.

### Whole tissue lysates

Samples were mechanically homogenized in RIPA buffer (50 mM Tris–HCl pH 7.4, 1% NP-40, 0.5% Na-deoxycholate, 0.1% SDS, 150 mM NaCl, 2 mM EDTA, 50 mM NaF) freshly supplemented with 0.5 mM PMSF, complete protease inhibitors (Roche) and phosphatase inhibitors (Roche) using a manual homogenizer. Samples were incubated on ice for 5 min and vortexed at maximum speed for 30 s. After centrifugation for 10 min at 3000 rpm and 4 °C the supernatant was used for further analyses. Protein amount was determined using DC protein assay from BioRad. Mitochondria were stored at  – 80 °C.

### Isolation of cardiac myocytes from mouse hearts

Ventricular myocytes were isolated using a modified Langendorff perfusion system. For anesthesia 5% isoflurane (Abbott GmbH & Co. KG) was used in combination with 250 I.U. unfractionated heparin and 0.33 mg carprofen. After loss of pedal reflexes, the heart was excised with the aortic arch, which was cannulated and perfused with prewarmed solutions at 37 °C. To remove blood from the coronary vessels, the heart was first perfused with solution A (113 mM NaCl, 4.7 mM KCl, 0.6 mM KH_2_PO_4_, 0.6 mM Na_2_HPO_4_, 1.2 mM MgSO_4_, 12 mM NaHCO_3_, 10 mM KHCO_3_, 10 mM HEPES, 32 µM Phenol red, 10 mM BDM, 30 mM Taurine, 5.5 mM Glucose) for 4 min. Next, the heart tissue was digested by perfusion with solution B (Solution A + 12.5 µM CaCl_2_, 0.08 mg/ml Liberase TH, 0.14 mg/ml Trypsin) for 5 to 8 min. To terminate the digestion the heart was removed from the Langendorff system, ventricles were opened and washed at room temperature with a 1:1 mixture of solution A and solution C (Solution A + 12.5 µM CaCl_2_, 10% FCS). To release intact cardiac myocytes, the heart was cautiously rinsed for 2–3 min with the mixture of solution A and C. The solution containing the cells was transferred to a 15 ml reaction tube and the cell pellet was obtained by sedimentation. The cells were resuspended in 5–10 mL solution D (Solution A + 12.5 µM CaCl_2_, 5% FCS) and transferred to a Petri dish. To restore the physiological concentration of extracellular Ca^2+^, five steps of 4 min each were performed with different CaCl_2_ concentrations (50 µM→ 100 µM→200 µM→500 µM→1 mM). Afterwards, the cells were transferred again to a 15 mL reaction tube again and the cell pellet was obtained by sedimentation for 8–10 min. The cells were finally resuspended in 10 mL culture medium (M199, 5% FCS, 1% penicillin and streptomycin, 10 mM HEPES) and kept at 37 °C.

### Analysis of gene expression

RNA was isolated from tissue with TRIzol™ Reagent (Thermo Fisher Scientific) or with the Monarch™ Total RNA Miniprep Kit (New England BioLabs) according to the manufacturers’ protocols. If genomic DNA digestion was not included in the RNA purification, the isolated RNA was further treated with RNase-free DNAse I (Thermo Fisher Scientific) according to the manufacturers’ protocol. RNA was transcribed in cDNA using the First Strand cDNA Synthesis Kit (Thermo Fisher Scientific) according to the manufacturers’ protocol. RT-qPCR was performed using the SensiMix SYBR Lo-ROX Kit (Bioline) and analyzed with the CXF96™ Real Time System (C1000 Touch Thermal Cycler, Bio rad). Used primers are indicated in the resources table.

### RNAseq analyses

Total RNA was isolated using RNAeasy mini kit (Qiagen) from frozen mouse heart and femur muscle. The library preparation and whole transcriptome sequencing were performed using Illumina platform by Novogene UK. Raw Fastq of 150 bp pair-end reads were mapped to mouse reference genome (mm10 GRCm38) using RNA STAR [24] with default settings. Raw Read counts were derived using tool FeatureCounts [57] v1.6.4. Differential expression analysis were then performed by DESeq2 [60] v2.11.40.6. DAVID tools v6.8 [44] was applied for gene functional enrichment analysis.

### Cell culture

WT and *Taz*^KO^ MEF cells, described in Chowdhury et al.[18], were cultivated in high-glucose DMEM (Gibco) supplemented with 100 U/mL penicillin/streptomycin (Gibco), 2 mM glutamine (Gibco) and 10% fetal calf serum (Sigma). Cells were kept at 37 °C and 5% CO_2_ in an incubator with constant air humidity. Cells were passaged every 2–3 days at 80–100% confluence. Cell number was determined by using the automated cell counter from BioRad. For knockdown experiments, transfection with siRNA was performed using Lipofectamine RNAiMAX transfection reagent (Invitrogen) according to manufacturer’s protocol. Confluent grown cells were incubated with the siRNA for 2 days. As a control, scrambled RNA was transfected the same way. Used siRNA sequences are listed in the resources table. To block the ISR pathway, cells were seeded to reach confluence after 1–2 days and incubated with 200 nM ISRIB overnight. For ^3^H-glutamine labeling, 20 kBq ^3^H-glutamine was added to Na^+^ free HBSS medium and ^3^H uptake was measured by scintillation counting. ^18^F-FASu uptake was measured in MEFs at different time points until 30 min. after administration of 100 kBq ^18^F-FASu to MEFs.

Development of iPSC from patient fibroblasts and characterization of differentiated cardiac myocytes has been described before [27]. iPSCs were cultured in mTeSRTM1 medium (BasalM + Supplement, STEMCELL) on prewarmed Matrigel (Corning) coated cell culture dishes at 37 °C, 5% CO_2_ supply and constant air humidity. Medium was changed every day. Upon reaching 100% confluence, cells were passaged using Accutase®-solution (Sigma) for detaching the cells. To stop Accutase®, DMEM/F12 medium (Gibco) was added. After each passaging step, the culture medium was supplemented with 10 µM ROCK inhibitor (Miltenyi Biotec). For differentiation, cells were seeded in 12-well plates with Matrigel coating and differentiation was induced at 80–90% confluence by changing the medium to RPMI 1640 (HEPES/ GlutaMax) medium (Gibco) supplemented with B27 without insulin (Gibco). 12 µM of CHIR99021 (GSK-3 Inhibitor XVI, Merk) was added to the cells for 24 h. After 48 h, 12 µM IWP2 (Wnt antagonist II, Merk) was added to the cells for 2 days. At day 7, the medium was changed to RPMI 1640 (HEPES/ GlutaMax) medium supplemented with B27 with insulin (Gibco) and medium was changed every other day from there on. For selection and enrichment of cardiac myocytes glucose within the medium was substituted with 4 mM lactate (Sigma) for 7 days.

Oxygen consumption rate (OCR) was measured using a Seahorse Extracellular flux Analyzer (Seahorse Bioscience, Billerica, MA, USA). Measurements were performed at basal levels, after the administration of 3 µM oligomycin, 1 µM FCCP, and 2 µM rotenone plus 1 µM antimycin A. Measurements of FAO using BSA coupled palmitate (167 µM final concentration for palmitate) were carried out in assay medium (111 mM NaCl, 4.7 mM KCl, 1.25 mM CaCl2, 2 mM MgSO4, 1.2 mM NaH2PO4) supplemented with 2.5 mM glucose, 0.5 mM carnitine, and 5 mM HEPES after starvation of cells in minimal medium (DMEM, 0.5 mM glucose, 1 mM glutamate, 1% FCS, 1 mM carnitine for 2 h). Measurements of glutamine metabolism in MEFs (20.000 cells/well) was performed in media supplemented with 1 mM pyruvate, 10 mM Glucose and 2 mM glutamine. Measurements were performed in at least eight technical replicates and repeated in independent experiments.

### Immunofluorescence staining

iPSC-derived cardiac myocytes were seeded 2 days before the experiment with a cell number of 10^5^ cells per well in six-well plates on cover slips with a diameter of 15 mm. Prior to the immunofluorescence staining, the cells were starved in HBSS solution for 24 h. Cells were incubated with 1 µM Bodipy (invitrogen) for 1 h at 37 °C. Afterwards, cells were washed once with PBS and incubated with 200 nM Mitotracker deep red (invitrogen) for 15 min at 37 °C. After two subsequently washing steps, cells were fixed with 4% PFA for 10 min and nuclei were stained with DAPI 500 ng/ml for 10 min. The cover slides were finally fixed on objective slides with Mowiol and cured for 30 min. Pictures were taken with the Leica DMi8 microscope at 63,000 × magnification.

### Enzymatic formate assay

To evaluate the formate production in mouse embryonal fibroblasts, the formate assay kit from Sigma Aldrich was used according to the manufacturer’s protocol. An additional protein determination of the sample lysates was performed using the DC protein assay from BioRad.

### Analysis of water-soluble metabolites in cell extracts

RP18 SPE-column (Phenomenex Strata C18-E, 55 µm, 50 mg / 1 ml) was activated by elution of 1 ml a CH_3_CN and afterwards equilibrated by elution of 1 ml MeOH/H_2_O (80/20, v/v). 490 µl 1 µM Lamivudine (Merck) dissolved MeOH/H_2_O (80/20, v/v) was added to dry cell pellet. Mixed samples were sonicated and after an additional mixing step they were centrifuged at maximum speed for 2 min. Supernatant was transferred to equilibrated RP18 SPE-column and the eluate was collected in a reaction tube. The residual metabolites were eluted using 150 µl MeOH/H_2_O (80/20 v/v) and the eluate was evaporated using a speed vacuum concentrator.

For liquid chromatography and mass spectrometry, the samples were dissolved again in 50 µl CH_3_CN/5 mM NH_4_OAc (25/75, v/v). For measurement, 3 µl sample was added to the ZIC-HILIC column (3.5 μm particles, 100 × 2.1 mm, Merck) combined with a SeQuant ZIC-HILIC pre-column (5 μm particles, 20 × 2 mm, Merck) and a Javelin particle Filter for 2.1 mm ID (Thermo Fisher Scientific). Column temperature was set to 30 °C and the LC gradient program started with 100% solvent B for 2 min, followed by a linear decrease to 40% solvent B within 16 min. Then, maintaining 40% B for 19 min and returning to 100% B in 2 min. before each injection column was equilibrated for 7 min with 100% solvent B. Two measurements were performed with mobile phase A consisting of 5 mM NH_4_OAc in CH_3_CN/H_2_O (5/95, v/v) and mobile phase B consisting of 5 mM NH_4_OAc in CH_3_CN/H_2_O (95/5, v/v) for the first measurement. In the second measurement, the mobile phase A consisted of MeOH/H_2_O/Formic Acid (5/94.9/0.1, v/v/v) and mobile phase B consisted of MeOH/H_2_O/Formic Acid (95/4.9/0.1, v/v/v). The flow rate was maintained at 200 μL/min and the eluent was directed to the ESI source of the QE-MS from 1.85 min to 20.0 min after sample injection. Measurements were performed with the Thermo Scientific Dionex Ultimate 3000 UHPLC system hyphenated with a Q Exactive mass spectrometer (QE-MS) equipped with a HESI probe (Thermo Fisher scientific). Peaks corresponding to the calculated amino acid masses (MIM + H +  ± 2 mMU) were integrated using TraceFinder software (Thermo Fisher Scientific).

### Analysis of water-soluble metabolites in heart tissue from in vivo isotope tracing

Tissue samples were pulverized using a Cryomill (Retsch). 10–20 mg of the resulting tissue powder was weighed and water-soluble metabolites were extracted with 40:40:20 methanol:acetonitrile:water with 0.5% formic acid pre-cooled to  – 20 °C. The ratio of extraction buffer volume to weighed tissue powder/volume of serum is 40:1. After vortexing for 10 s and keeping samples in ice for 10 min, samples were neutralized by adding 15% ammonium bicarbonate (NH_4_HCO_3_) aqueous solution (8.75% v/v of extraction buffer). Samples were then again mixed by vortexing for 10 s, centrifuged at 16,000 × g for 30 min at 4 °C, and then supernatant was transferred to LC–MS vials for analysis. Water-soluble metabolite measurements were obtained by running samples on the Q Exactive PLUS hybrid quadrupole-orbitrap mass spectrometer (Thermo Scientific) coupled with hydrophilic interaction chromatography (HILIC). We use XBridge BEH Amide column (150 mm × 2.1 mm, 2.5 μM particle size, Waters, Milford, MA). Solvent A (95%:5% H_2_O:acetonitrile with 20 mM ammonium acetate, 20 mM ammonium hydroxide, pH 9.4) and solvent B (100% acetonitrile) is running with gradient: 0 min, 90% B; 2 min, 90% B; 3 min, 75%; 7 min, 75% B; 8 min, 70%, 9 min, 70% B; 10 min, 50% B; 12 min, 50% B; 13 min, 25% B; 14 min, 25% B; 16 min, 0% B, 20.5 min, 0% B; 21 min, 90% B; 25 min, 90% B. The injection volume is 5 μL, the flow rate was 150 μL/min with a column temperature of 25 °C. The MS scans were in negative ion mode with a resolution of 140,000 at m/z 200. All data from isotope labeling experiments were analyzed by El-MAVEN with natural abundance correction.

### Statistics

If not indicated otherwise non-paired Student´s *t*-test was performed for statistical analysis using the Graph Pad 7.05 Software. Results are displayed as mean and SEM if not indicated otherwise.

### Miscellaneous

For SDS-Page and western blot, standard protocols were applied. In short, whole tissue lysates or isolated mitochondria were loaded onto acrylamide gels. Acrylamide concentrations were chosen between 10% and 12.5% depending on the desired protein size. Proteins were transferred onto a PVDF membrane (Immobilon-P 0.45 µM pore size, Merck Millipore) via semi-dry blot technique. The membrane was incubated in 5% non-fat dry milk dissolved in TBST (200 mM Tris/HCl pH 7.4, 1.25 M NaCl, 0.1% Tween 20) for 1 h at room temperature or at 4 °C over night. The incubation in the primary antibody was performed over night at 4 °C or at room temperature for at least 1 h. Used primary and secondary antibodies are indicated in resources table. Secondary antibodies were directed against rabbit or mouse and conjugated with horseradish peroxidase. Protein signals were visualized with ECL™ Western Blotting Detection Reagents (GE Healthcare Amersham™) using the ChemiDocTM Imager (BioRad). Quantification of the western blot signals was done using the software Image Lab 6.0 (Bio Rad).

### Supplementary Information

Below is the link to the electronic supplementary material.

Supplementary file1 (PDF 734 KB) Supplementary Fig. 1 Fatty acid oxidation defect in Taz-KD mouse model. A Body weight of WT and Taz-KD mouse model. B Heart weight/ body weight ratio of WT and Taz-KD mouse model. C Muscle weight of WT and Taz-KD mouse model. D Blood glucose levels in WT and Taz-KD mouse (n=5 for panels A-D). E Analysis of gene expression of indicated glucose transporters in Taz-KD mouse heart by qPCR normalized to Actb, (β-actin, n=9 for Glut1, n=8 for Glut4, n=3 for Glut8). F Octanoylcarnitine driven oxygen consumption of isolated heart mitochondria from Taz-KD mice and control in absence (state 2) and presence (state3) of ADP. Respiratory measurements was followed by oligomycin inhibition of the F1FO ATPase and subsequent DNP mediated uncoupling of the membrane potential. G Quantification of western blots in Fig. 1E (n=3 per genotype). H Western blot analysis of TMLHE protein levels compared to VDAC in heart tissue lysates from WT and Taz-KD mice. I Analysis of TMLHE gene expression in Taz-KD mouse heart by qPCR normalized to Gapdh (n=3). J Analysis of Acsl gene expression in Taz-KD mouse heart by qPCR normalized to mS12 (n=4). K Analysis of O-GlcNAc-ylation of proteins in tissue lysate of mouse heart by western blot and subsequent quantification, normalized to TUBULIN as a control Taz-KD vs. WT (n=5). L Western blot analysis of PINK1 accumulation on isolated mitochondria. M Quantification of PINK1 and VDAC, analyzed by western blot of isolated mitochondria (n=6). N Quantification of LC3-I and the processed form LC3-II in western blot analysis of cardiac lysates (WT, n=10; Taz-KD, n=11). O Analysis of gene expression of Bcl-2 related to Actb (β-actin) as a control in mouse heart samples (n= 7). Data represent mean ± SEM; n-numbers are indicated as numbers of animals. Statistical significance was determined with unpaired Student’s t-test in panels A, E, G, I, J, M and O and by 2-way ANOVA followed by Bonferroni post-test for panel F. IOD, integrated optic density

Supplementary file2 (PDF 363 KB) Supplementary Fig. 2 Defect in mitochondrial transport and oxidation in TAZ10 iPSC-CM. A OCR of iPSC-CM from one BTHS patient (TAZ10) and one healthy control supplied with BSA-coupled palmitate, n=2, representative experiment shown. AntA, Antimycin A. B Analysis of gene expression in iPSC derived cardiac myocytes of control and TAZ10 by qPCR using primers against indicated mRNA. Data normalized to the mitochondrial ribosomal protein L28. (n>/=3). C Representative images of TAZ10 and control iPSC-derived cardiac myocytes supplied with Bodipy-labeled fatty acids. Mitochondria were stained with Mitotracker (red) and nuclei were stained with DAPI (blue). D Manual counting of cells exhibiting overlay of Bodipy-labeled fatty acids and mitochondrial signals, indicative of translocation events with all counted cells normalized to 100 cells. E Automated determination of the ratio of areas from lipid droplets (Bodipy) and mitochondria (Mitotracker), n=21-31 cells. Data represent mean ± SEM; n-numbers are indicated as numbers of independent experiments and statistical significance was determined with unpaired Student’s t-test in panel B

Supplementary file3 (PDF 411 KB) Supplementary Fig. 3 Fatty acid oxidation defect in skeletal muscle of BTHS mice. A Uptake of 18F-FDG into gastrocnemius muscle (n=5 for WT and n=4 for Taz-KD). B 18F-FTOa uptake into gastrocnemius muscle (n=5 per genotype). C Oxygen consumption of isolated skeletal muscle mitochondria from Taz-KD mice and control. Respiration is induced by administration of pyruvate and malate. Respiration was measured in absence (state 2) and presence (state3) of ADP. Respiratory measurements was followed by oligomycin inhibition of F1FO ATPase and subsequent DNP mediated uncoupling of membrane potential. D Palmitoyl-CoA respiration of isolated mitochondria under conditions as in (C). E Respiration of isolated mitochondria on palmitoylcarnitine under conditions as in (C). F Western blot analysis of indicated protein levels from skeletal muscle tissue lysates from WT and Taz-KD mice compared to VDAC. G Analysis of gene expression in skeletal muscle tissue by qPCR using primers against indicated mRNAs displayed as fold change (FC) of WT normalized to mS12. n=3. Data represent mean ± SEM; n-numbers are indicated as numbers of animals. Statistical significance was determined by 2-way ANOVA followed by Bonferroni post-test for panel C, D and E

Supplementary file4 (PDF 268 KB) Supplementary Fig. 4 Transcriptome analysis of cardiac tissue. A Heatmap showing the cardiac gene expression profile in Taz-KD vs. WT control. The genes are clustered with hierarchical clustering using Pearson correlation by Cluser3.0 [22]. Normalized expression level were derived from Deseq2. B Functional enrichment analysis (GO terms for biological process) for the upregulated genes in heart tissue. 262 upregulated coding genes with fold change >2 was used. Top 30 functions/pathways that are enriched in GO Biological Process of the significantly upregulated genes (FC>2). Bar length indicates the log transformed p-value from Fisher exact test

Supplementary file5 (PDF 594 KB) Supplementary Fig. 5 Enzymes of serine and 1C metabolism are upregulated in BTHS. A Densitometry analysis of PHGDH and SHMT1 protein levels, compared to GAPDH in mouse cardiac lysates (n=4). B Quantifications of western blots in Fig. 3F. C Densitometry of western blots in Fig. 3G. D Quantification of western blots of MTHFD2 in cardiac lysates compared to VDAC as a control (n=5). E Analysis of gene expression in cardiac tissue by qPCR using primers against Shmt1 using Gapdh as a control. F qPCR as in E with primers against Shmt2. G Quantification of gene expression of Cpt2 (left panel) and Mthfd2 (right panel) using mS12 as a control in 10 weeks old mice. H Analysis as in (G) with 30 weeks old mice. I Quantification of western blot analysis of CPT2 compared to VDAC in isolated cardiac mitochondria of 10 weeks old mice (n=4-5). J Western blot of indicated proteins in isolated cardiac mitochondria in 30 weeks old Taz-KD mice (n=3). K Densitometry of western blots shown in Fig. 2L. Data represent mean ± SEM; n-numbers are indicated as numbers of animals for panels A-F. Statistical significance was determined with unpaired Student’s t-test in panels A-D, F, G, H and I

Supplementary file6 (PDF 388 KB) Supplementary Fig. 6 ISR activation in heart and skeletal muscle. A Quantitative densitometry of western blots in Fig. 5A. B Quantifications of western blots for ATF4 compared to levels of TUBULIN as a control in Taz-KD mouse hearts (n=6). C Western blot analysis of phosphorylated and non-phosphorylated PERK (n=3). D Heatmap showing gene expression profiles in skeletal muscle of Taz-KD vs. WT control. The genes are clustered with hierarchical clustering using Pearson correlation by Cluser3.0 [22]. Normalized expression level were derived from Deseq2. E Overlap in gene pattern in transcriptome analysis of cardiac tissue and skeletal muscle tissue of Taz-KD mice. F Volcano plot of genes in Taz-KD skeletal muscle in comparison with WT. X-axis: log2 transformed fold changes. Y-axis: minus log10 transformed p value. G qPCR analysis of gene expression of indicated mRNAs from skeletal muscle normalized to Gapdh. Data are displayed as fold change (FC) of WT (n=3). H Western blot analysis of MTHFD2 in isolated mitochondria of skeletal muscle tissue from WT and three Taz-KD mice (n=3). Data represent mean ± SEM; n-numbers are indicated as numbers of animals. Statistical significance in panels A, B, and G was determined by unpaired Student’s t-test

Supplementary file7 (PDF 396 KB) Supplementary Fig. 7 Genetic ablation of Taz induces Atf4 and Gadd45 gene expression. A Quantification of western blot analysis, shown in Figure 5A of ATF4 in TazKO and WT MEFs (n=3). B Western blot analysis of indicated proteins in WT and TazKO MEFs treated or not with tunicamycin (Tm). C Quantification of three additional experiments as in B, normalized to WT n.t. D Western blot analysis of WT and TazKO MEF cell lysates for PSAT1 compared to GAPDH. n=3. E qPCR analysis of Atf4 gene expression in siRNA treated MEFs against Atf4 or control. n=5. F qPCR analysis of Gadd45 gene expression in siGadd45 or control treated MEFs, n=5. G qPCR analysis of gene expression of Glut1 (left panel) and Glut4 (right panel) in TazKO MEFs, treated with ISRIB. H Quantification of protein O-GlcNAc-ylation in cell lysates of TazKO MEFs (n=5). I Quantification of LC3-II to LC3-I ratio by western blot analysis of MEF lysates and Bafilomycin treated cells as a control (n=3). J Quantification of LC3-II to LC3-I ratio in MEF, treated with ISRIB (n=4). K Analysis of surviving cells after glucose starvation normalized not starved cells (n.t.; n=4). Data represent mean ± SEM; n-numbers represents individual experiments. For statistical analysis of Fig. A, B, F, H, I and J and a t-test and for Fig. D and E a one way ANOVA followed by Tuckey´s multiple comparisons test was performed

Supplementary file8 (PDF 409 KB) Supplementary Fig. 8 Genes involved in amino acid metabolism in Taz-KD mouse heart. A qPCR analysis of indicated genes related to Gapdh as a control. n=3. B qPCR analysis of Gpt2 related to Gapdh as a control. n=3. C Oxygen consumption rate (OCR) of MEFs supplied with glutamine and treated with aminooxyacetate (AOA) or DMSO. AntA, Antimycin A. D Quantification of five experiments as shown in (C) with basal respiration normalized to n.t. control for both genotypes. n=5. E Respiration (Oxygen consumption rate, OCR) of MEFs supplied in glutamine containing medium and treated with a specific inhibitor of the kinase PERK (PERKi) or DMSO. F Quantification of three experiments as shown in (E) with basal respiration normalized to n.t. control for both genotypes. n=3. G Gene expression of Pck2 related to Gapdh as a control, n=6 for WT and n=5 for Taz-KD. H qPCR analysis of Pycr1 related to Gapdh as a control. n=5. I Quantification of western blot analysis of P5CS (gene product of Aldh18a1), normalized to VDAC as a control (n=5). Data represent mean ± SEM; n-numbers are indicated as numbers of animals. Statistical significance was determined by unpaired Student’s t-test

Supplementary file9 (PDF 400 KB) Supplementary Fig. 9 Slc7a11 gene expression in skeletal muscle and MEF cells. A Analysis of indicated metabolites by mass-spectrometry. Relative amounts of metabolites, normalized to total metabolites with WT set to 100%. B qPCR analysis of Slc7a11 gene expression in skeletal muscle tissue from WT and Taz-KD mice displayed as fold change (FC) of WT (n=3, N.D. = not detectable). C qPCR analysis of Slc7a11 gene expression in MEF cells. n=3. D qPCR analysis of Slc7a11 gene expression in MEF cells treated with siRNA against Atf4 or control normalized to Gapdh. n=3. E Western blot analysis of GPX4 and GAPDH in cardiac lysates of Taz-KD vs. WT mice (left panel) and quantification (right panel, n=3). F qPCR analysis of Gpx1 in MEF cells treated with ISRIB or DMSO as control normalized to mS12. n=5. Data represent mean ± SEM; n-numbers represent individual animals in panels B and E, independent cell experiments in A, C, D and D- F. Statistical significance was determined with unpaired Student’s t-test in panel A,C and E. For statistical analysis of panel D and F, a one way ANOVA followed by Tuckey´s multiple comparisons test was performed

Supplementary file10 (DOCX 17 KB)

## Data Availability

Raw transcriptomic data are available upon request.

## Abbreviations

1C: One carbon metabolism
BTHS: Barth Syndrome
CL: Cardiolipin
DCM: Dilated cardiomyopathy
DNP: 2,4-Dinitrophenol
ER: Endoplasmic reticulum
FA: Fatty acid
GSH: Glutathione
GPX: Glutathione peroxidase
IOD: Integrated optic density
ISR: Integrated stress response
KD: Knockdown
LVAD: Left ventricular assist device
MEF: Mouse embryonic fibroblast
OCR: Oxygen consumption rate
PCR: Polymerase chain reaction
ROS: Reactive oxygen species
*Taz*: Tafazzin gene
*Taz*-KD: Inducible TAZ knockdown mice
TIC: Total ion chromatogram
WT: Wild type
