# Supplementary figures and images for: Activation of the integrated stress response rewires cardiac metabolism in Barth syndrome
Source: Basic Res Cardiol. 2023 Nov 6;118(1):47. doi: 10.1007/s00395-023-01017-x (PMC10628049; doi:10.1007/s00395-023-01017-x)

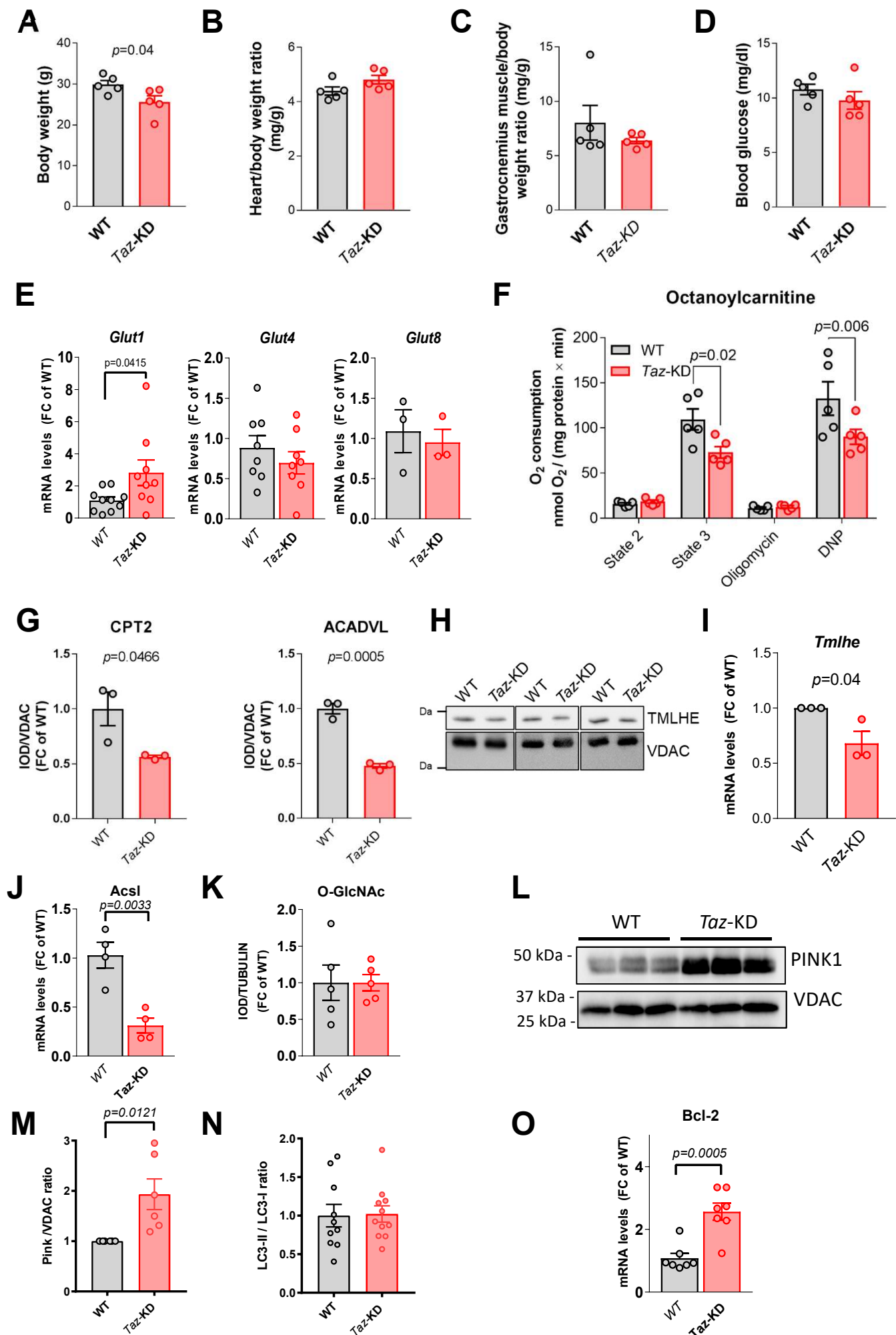

Supplement: Supplementary file 1 — Supplementary file1 (PDF 734 KB) Supplementary Fig. 1 Fatty acid oxidation defect in Taz-KD mouse model. A Body weight of WT and Taz-KD mouse model. B Heart weight/ body weight ratio of WT and Taz-KD mouse model. C Muscle weight of WT and Taz-KD mouse model. D Blood glucose levels in WT and Taz-KD mouse (n=5 for panels A-D). E Analysis of gene expression of indicated glucose transporters in Taz-KD mouse heart by qPCR normalized to Actb, (β-actin, n=9 for Glut1, n=8 for Glut4, n=3 for Glut8). F Octanoylcarnitine driven oxygen consumption of isolated heart mitochondria from Taz-KD mice and control in absence (state 2) and presence (state3) of ADP. Respiratory measurements was followed by oligomycin inhibition of the F1FO ATPase and subsequent DNP mediated uncoupling of the membrane potential. G Quantification of western blots in Fig. 1E (n=3 per genotype). H Western blot analysis of TMLHE protein levels compared to VDAC in heart tissue lysates from WT and Taz-KD mice. I Analysis of TMLHE gene expression in Taz-KD mouse heart by qPCR normalized to Gapdh (n=3). J Analysis of Acsl gene expression in Taz-KD mouse heart by qPCR normalized to mS12 (n=4). K Analysis of O-GlcNAc-ylation of proteins in tissue lysate of mouse heart by western blot and subsequent quantification, normalized to TUBULIN as a control Taz-KD vs. WT (n=5). L Western blot analysis of PINK1 accumulation on isolated mitochondria. M Quantification of PINK1 and VDAC, analyzed by western blot of isolated mitochondria (n=6). N Quantification of LC3-I and the processed form LC3-II in western blot analysis of cardiac lysates (WT, n=10; Taz-KD, n=11). O Analysis of gene expression of Bcl-2 related to Actb (β-actin) as a control in mouse heart samples (n= 7). Data represent mean ± SEM; n-numbers are indicated as numbers of animals. Statistical significance was determined with unpaired Student’s t-test in panels A, E, G, I, J, M and O and by 2-way ANOVA followed by Bonferroni post-test for panel F. IOD, integrate [file 395_2023_1017_MOESM1_ESM.pdf]

**A**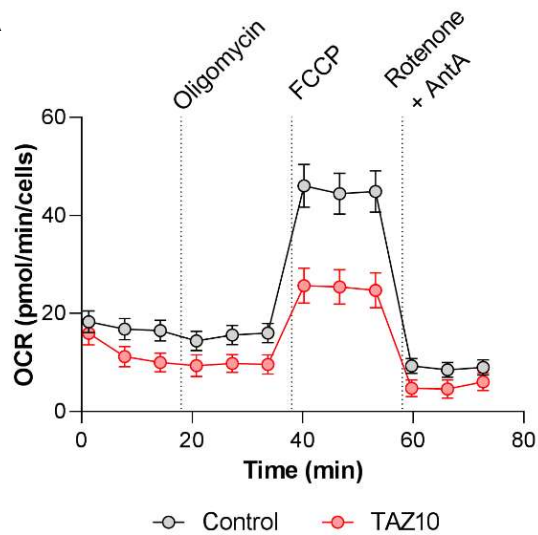**B**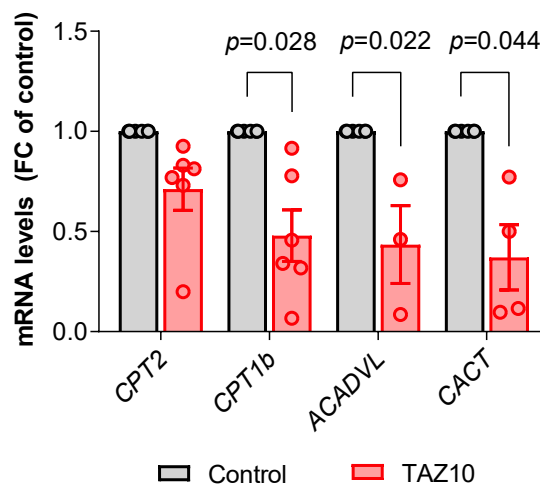**C**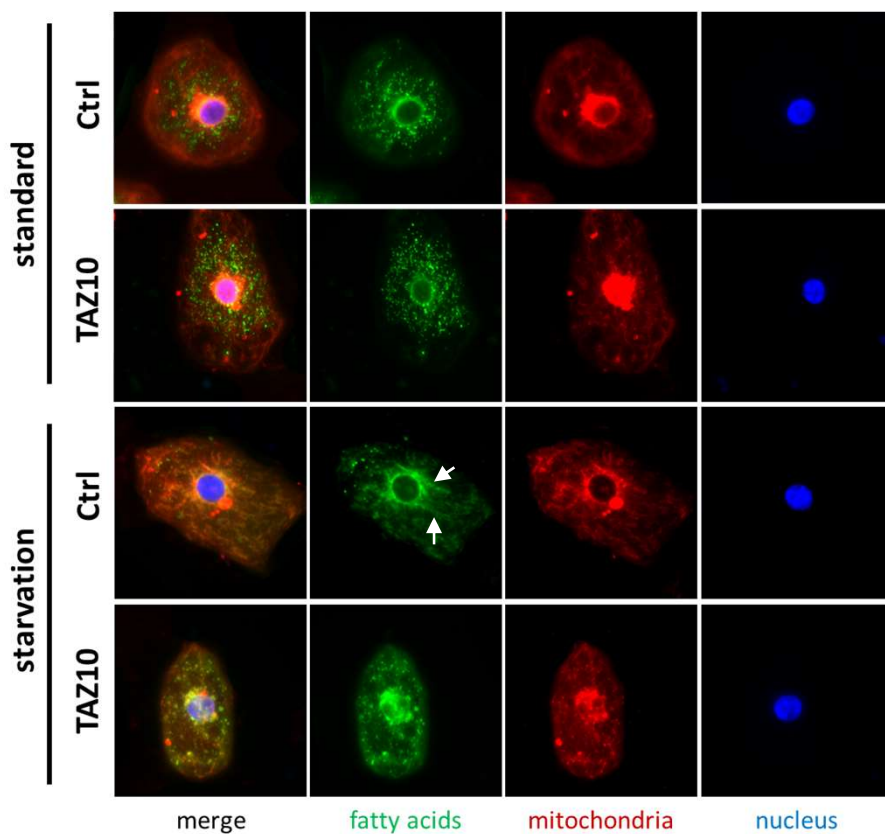**D**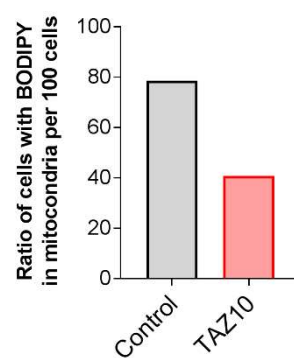**E**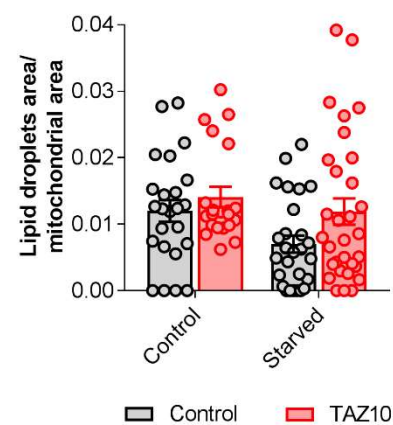

Supplement: Supplementary file 2 — Supplementary file2 (PDF 363 KB) Supplementary Fig. 2 Defect in mitochondrial transport and oxidation in TAZ10 iPSC-CM. A OCR of iPSC-CM from one BTHS patient (TAZ10) and one healthy control supplied with BSA-coupled palmitate, n=2, representative experiment shown. AntA, Antimycin A. B Analysis of gene expression in iPSC derived cardiac myocytes of control and TAZ10 by qPCR using primers against indicated mRNA. Data normalized to the mitochondrial ribosomal protein L28. (n>/=3). C Representative images of TAZ10 and control iPSC-derived cardiac myocytes supplied with Bodipy-labeled fatty acids. Mitochondria were stained with Mitotracker (red) and nuclei were stained with DAPI (blue). D Manual counting of cells exhibiting overlay of Bodipy-labeled fatty acids and mitochondrial signals, indicative of translocation events with all counted cells normalized to 100 cells. E Automated determination of the ratio of areas from lipid droplets (Bodipy) and mitochondria (Mitotracker), n=21-31 cells. Data represent mean ± SEM; n-numbers are indicated as numbers of independent experiments and statistical significance was determined with unpaired Student’s t-test in panel B [file 395_2023_1017_MOESM2_ESM.pdf]

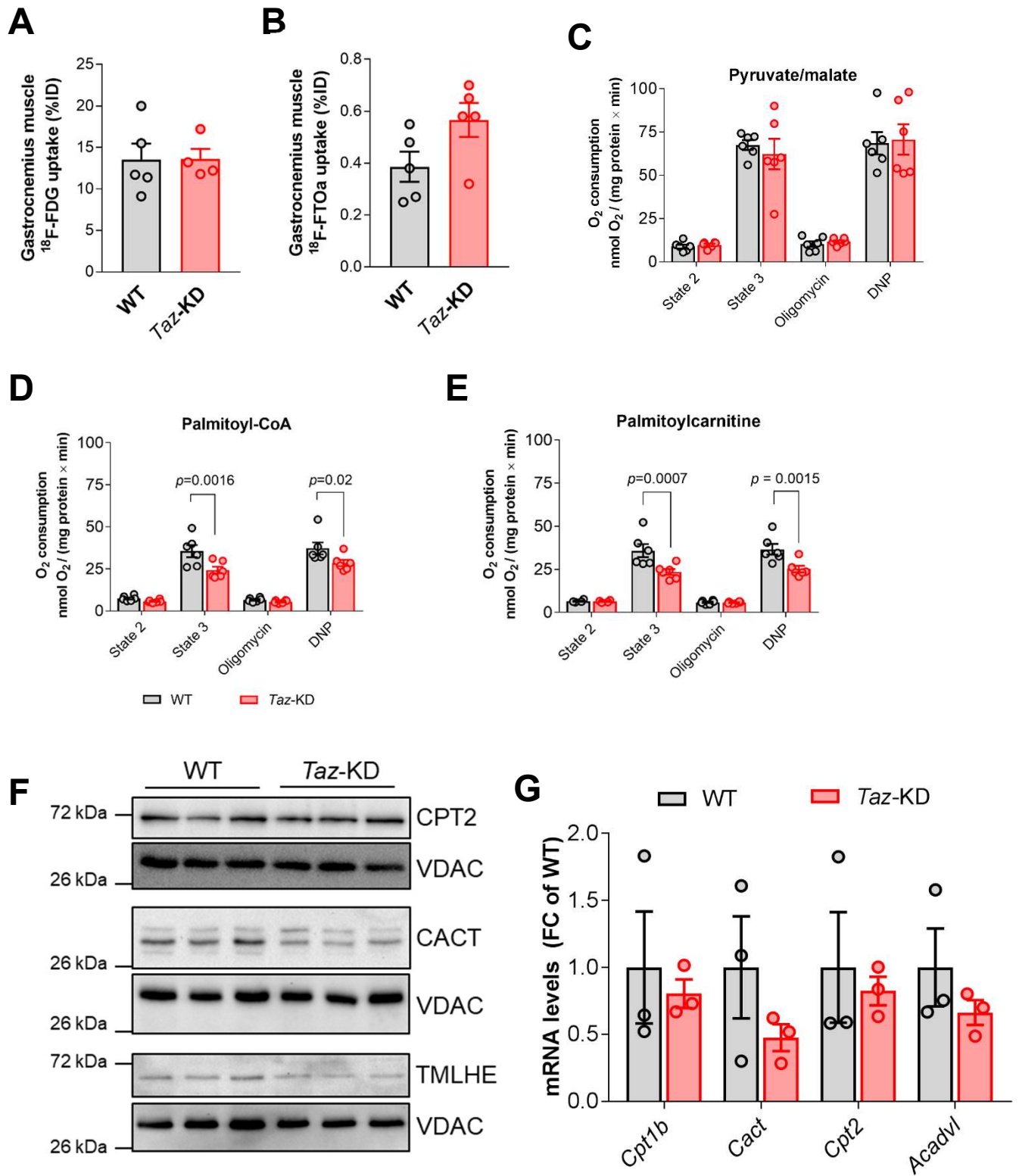

Supplement: Supplementary file 3 — Supplementary file3 (PDF 411 KB) Supplementary Fig. 3 Fatty acid oxidation defect in skeletal muscle of BTHS mice. A Uptake of 18F-FDG into gastrocnemius muscle (n=5 for WT and n=4 for Taz-KD). B 18F-FTOa uptake into gastrocnemius muscle (n=5 per genotype). C Oxygen consumption of isolated skeletal muscle mitochondria from Taz-KD mice and control. Respiration is induced by administration of pyruvate and malate. Respiration was measured in absence (state 2) and presence (state3) of ADP. Respiratory measurements was followed by oligomycin inhibition of F1FO ATPase and subsequent DNP mediated uncoupling of membrane potential. D Palmitoyl-CoA respiration of isolated mitochondria under conditions as in (C). E Respiration of isolated mitochondria on palmitoylcarnitine under conditions as in (C). F Western blot analysis of indicated protein levels from skeletal muscle tissue lysates from WT and Taz-KD mice compared to VDAC. G Analysis of gene expression in skeletal muscle tissue by qPCR using primers against indicated mRNAs displayed as fold change (FC) of WT normalized to mS12. n=3. Data represent mean ± SEM; n-numbers are indicated as numbers of animals. Statistical significance was determined by 2-way ANOVA followed by Bonferroni post-test for panel C, D and E [file 395_2023_1017_MOESM3_ESM.pdf]

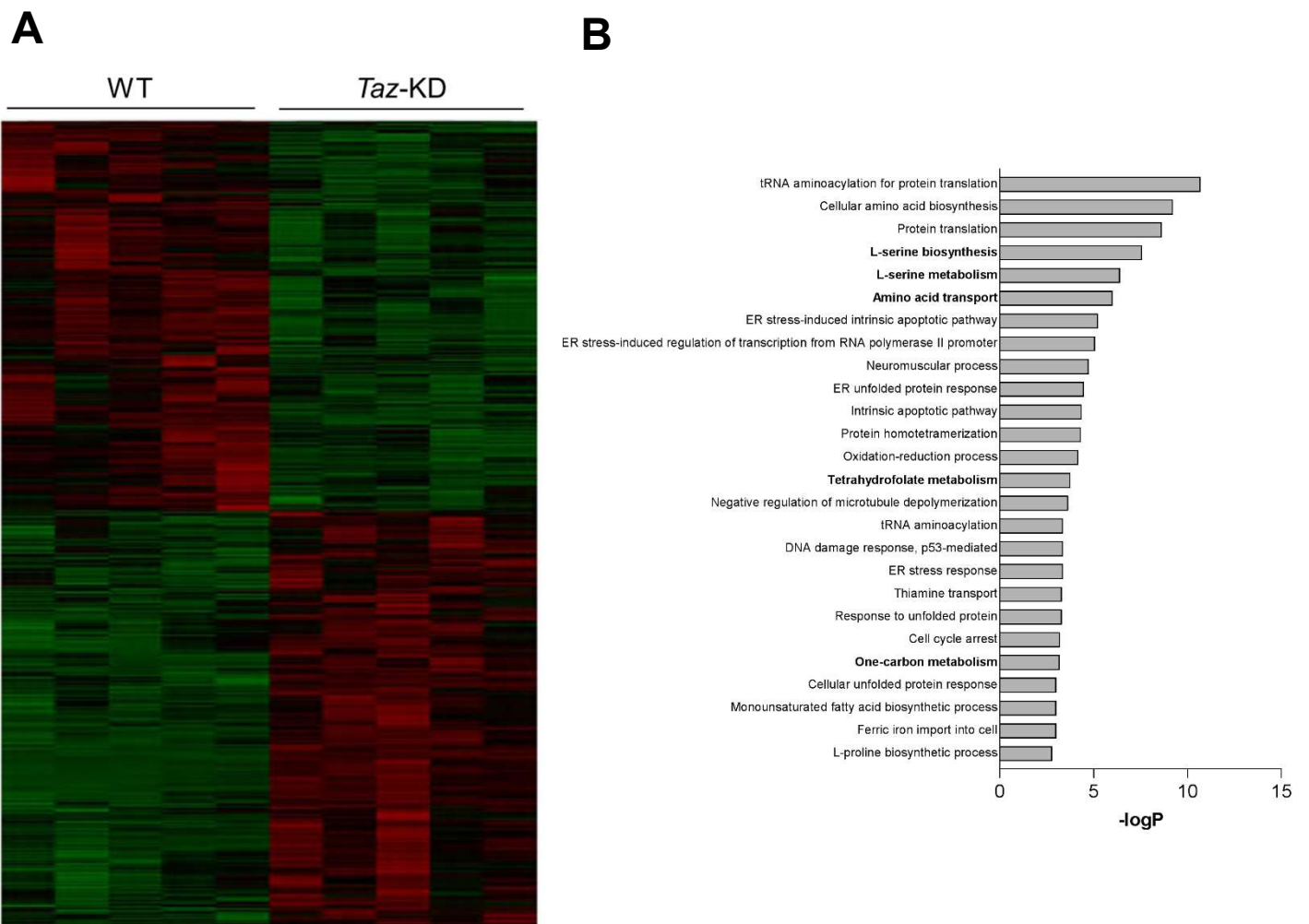

Supplement: Supplementary file 4 — Supplementary file4 (PDF 268 KB) Supplementary Fig. 4 Transcriptome analysis of cardiac tissue. A Heatmap showing the cardiac gene expression profile in Taz-KD vs. WT control. The genes are clustered with hierarchical clustering using Pearson correlation by Cluser3.0 [22]. Normalized expression level were derived from Deseq2. B Functional enrichment analysis (GO terms for biological process) for the upregulated genes in heart tissue. 262 upregulated coding genes with fold change >2 was used. Top 30 functions/pathways that are enriched in GO Biological Process of the significantly upregulated genes (FC>2). Bar length indicates the log transformed p-value from Fisher exact test [file 395_2023_1017_MOESM4_ESM.pdf]

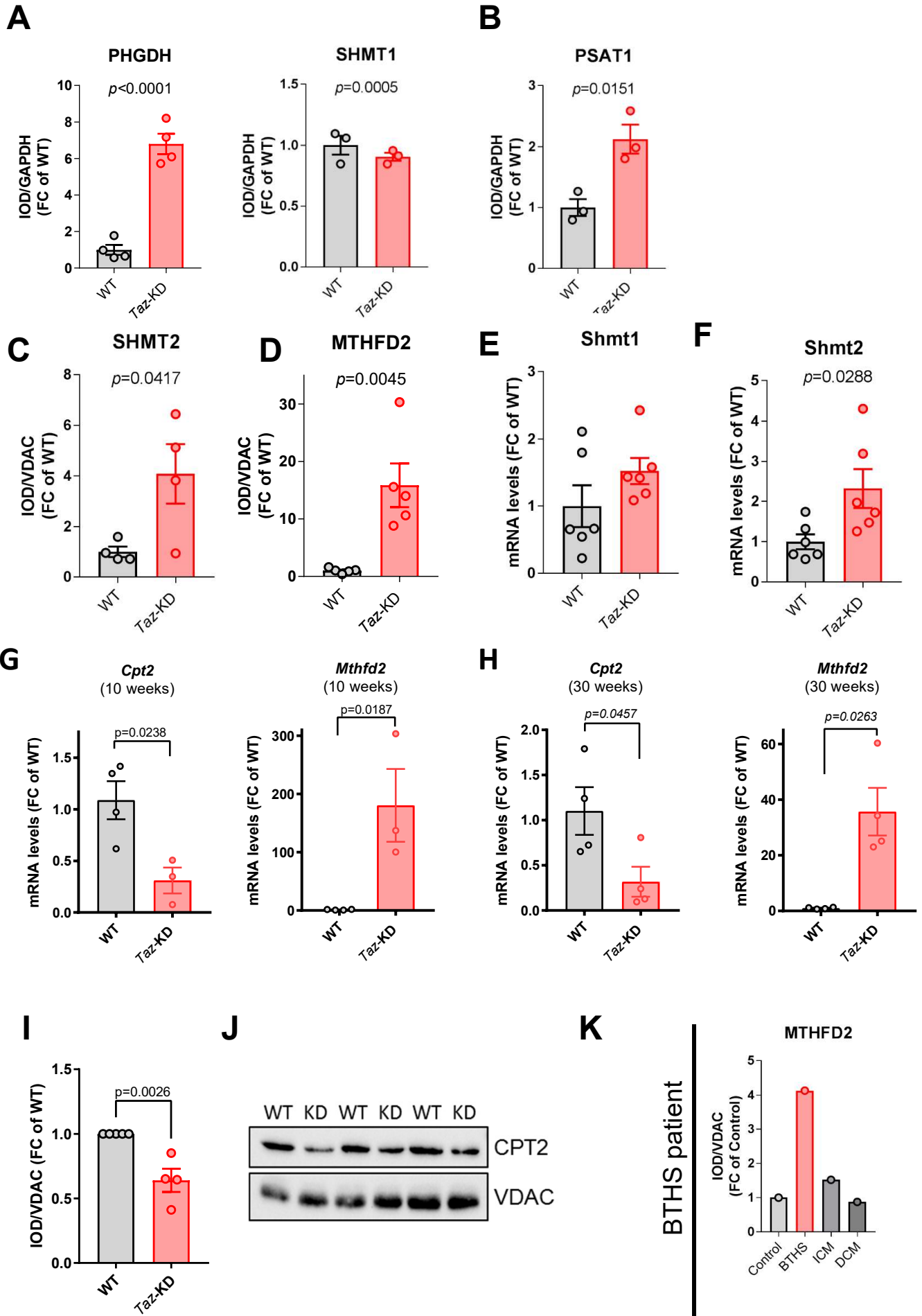

Supplement: Supplementary file 5 — Supplementary file5 (PDF 594 KB) Supplementary Fig. 5 Enzymes of serine and 1C metabolism are upregulated in BTHS. A Densitometry analysis of PHGDH and SHMT1 protein levels, compared to GAPDH in mouse cardiac lysates (n=4). B Quantifications of western blots in Fig. 3F. C Densitometry of western blots in Fig. 3G. D Quantification of western blots of MTHFD2 in cardiac lysates compared to VDAC as a control (n=5). E Analysis of gene expression in cardiac tissue by qPCR using primers against Shmt1 using Gapdh as a control. F qPCR as in E with primers against Shmt2. G Quantification of gene expression of Cpt2 (left panel) and Mthfd2 (right panel) using mS12 as a control in 10 weeks old mice. H Analysis as in (G) with 30 weeks old mice. I Quantification of western blot analysis of CPT2 compared to VDAC in isolated cardiac mitochondria of 10 weeks old mice (n=4-5). J Western blot of indicated proteins in isolated cardiac mitochondria in 30 weeks old Taz-KD mice (n=3). K Densitometry of western blots shown in Fig. 2L. Data represent mean ± SEM; n-numbers are indicated as numbers of animals for panels A-F. Statistical significance was determined with unpaired Student’s t-test in panels A-D, F, G, H and I [file 395_2023_1017_MOESM5_ESM.pdf]

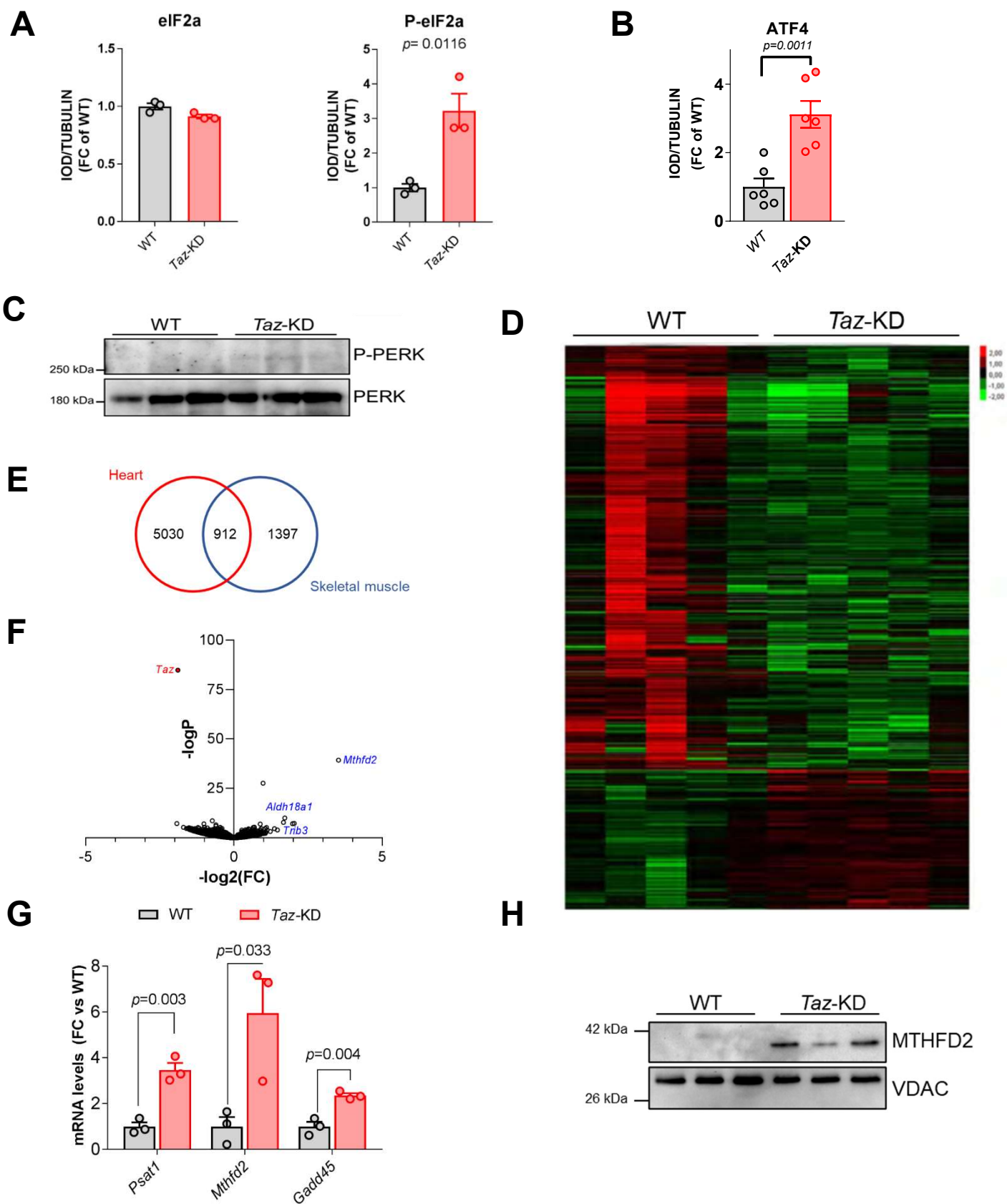

Supplement: Supplementary file 6 — Supplementary file6 (PDF 388 KB) Supplementary Fig. 6 ISR activation in heart and skeletal muscle. A Quantitative densitometry of western blots in Fig. 5A. B Quantifications of western blots for ATF4 compared to levels of TUBULIN as a control in Taz-KD mouse hearts (n=6). C Western blot analysis of phosphorylated and non-phosphorylated PERK (n=3). D Heatmap showing gene expression profiles in skeletal muscle of Taz-KD vs. WT control. The genes are clustered with hierarchical clustering using Pearson correlation by Cluser3.0 [22]. Normalized expression level were derived from Deseq2. E Overlap in gene pattern in transcriptome analysis of cardiac tissue and skeletal muscle tissue of Taz-KD mice. F Volcano plot of genes in Taz-KD skeletal muscle in comparison with WT. X-axis: log2 transformed fold changes. Y-axis: minus log10 transformed p value. G qPCR analysis of gene expression of indicated mRNAs from skeletal muscle normalized to Gapdh. Data are displayed as fold change (FC) of WT (n=3). H Western blot analysis of MTHFD2 in isolated mitochondria of skeletal muscle tissue from WT and three Taz-KD mice (n=3). Data represent mean ± SEM; n-numbers are indicated as numbers of animals. Statistical significance in panels A, B, and G was determined by unpaired Student’s t-test [file 395_2023_1017_MOESM6_ESM.pdf]

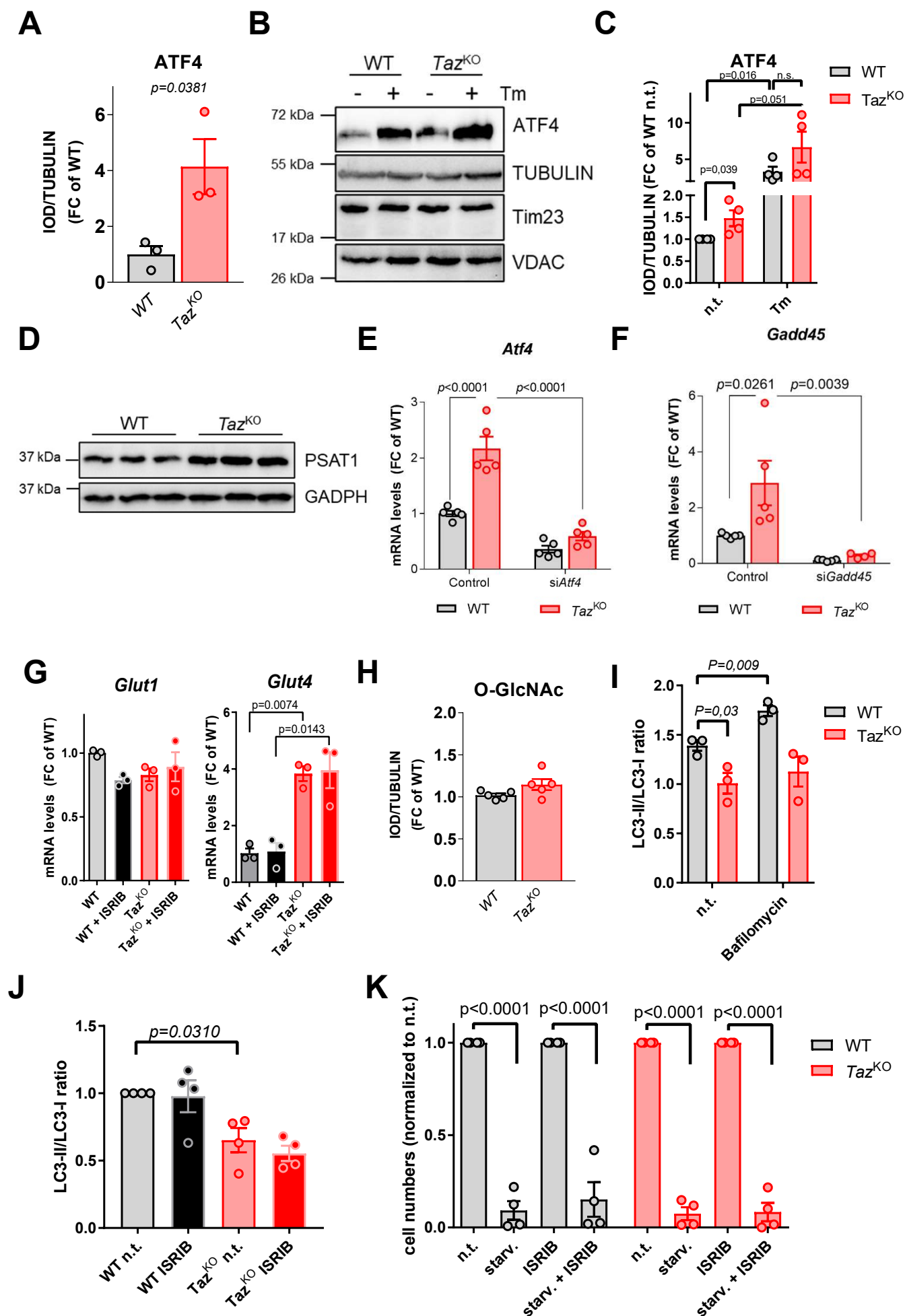

Supplement: Supplementary file 7 — Supplementary file7 (PDF 396 KB) Supplementary Fig. 7 Genetic ablation of Taz induces Atf4 and Gadd45 gene expression. A Quantification of western blot analysis, shown in Figure 5A of ATF4 in TazKO and WT MEFs (n=3). B Western blot analysis of indicated proteins in WT and TazKO MEFs treated or not with tunicamycin (Tm). C Quantification of three additional experiments as in B, normalized to WT n.t. D Western blot analysis of WT and TazKO MEF cell lysates for PSAT1 compared to GAPDH. n=3. E qPCR analysis of Atf4 gene expression in siRNA treated MEFs against Atf4 or control. n=5. F qPCR analysis of Gadd45 gene expression in siGadd45 or control treated MEFs, n=5. G qPCR analysis of gene expression of Glut1 (left panel) and Glut4 (right panel) in TazKO MEFs, treated with ISRIB. H Quantification of protein O-GlcNAc-ylation in cell lysates of TazKO MEFs (n=5). I Quantification of LC3-II to LC3-I ratio by western blot analysis of MEF lysates and Bafilomycin treated cells as a control (n=3). J Quantification of LC3-II to LC3-I ratio in MEF, treated with ISRIB (n=4). K Analysis of surviving cells after glucose starvation normalized not starved cells (n.t.; n=4). Data represent mean ± SEM; n-numbers represents individual experiments. For statistical analysis of Fig. A, B, F, H, I and J and a t-test and for Fig. D and E a one way ANOVA followed by Tuckey´s multiple comparisons test was performed [file 395_2023_1017_MOESM7_ESM.pdf]

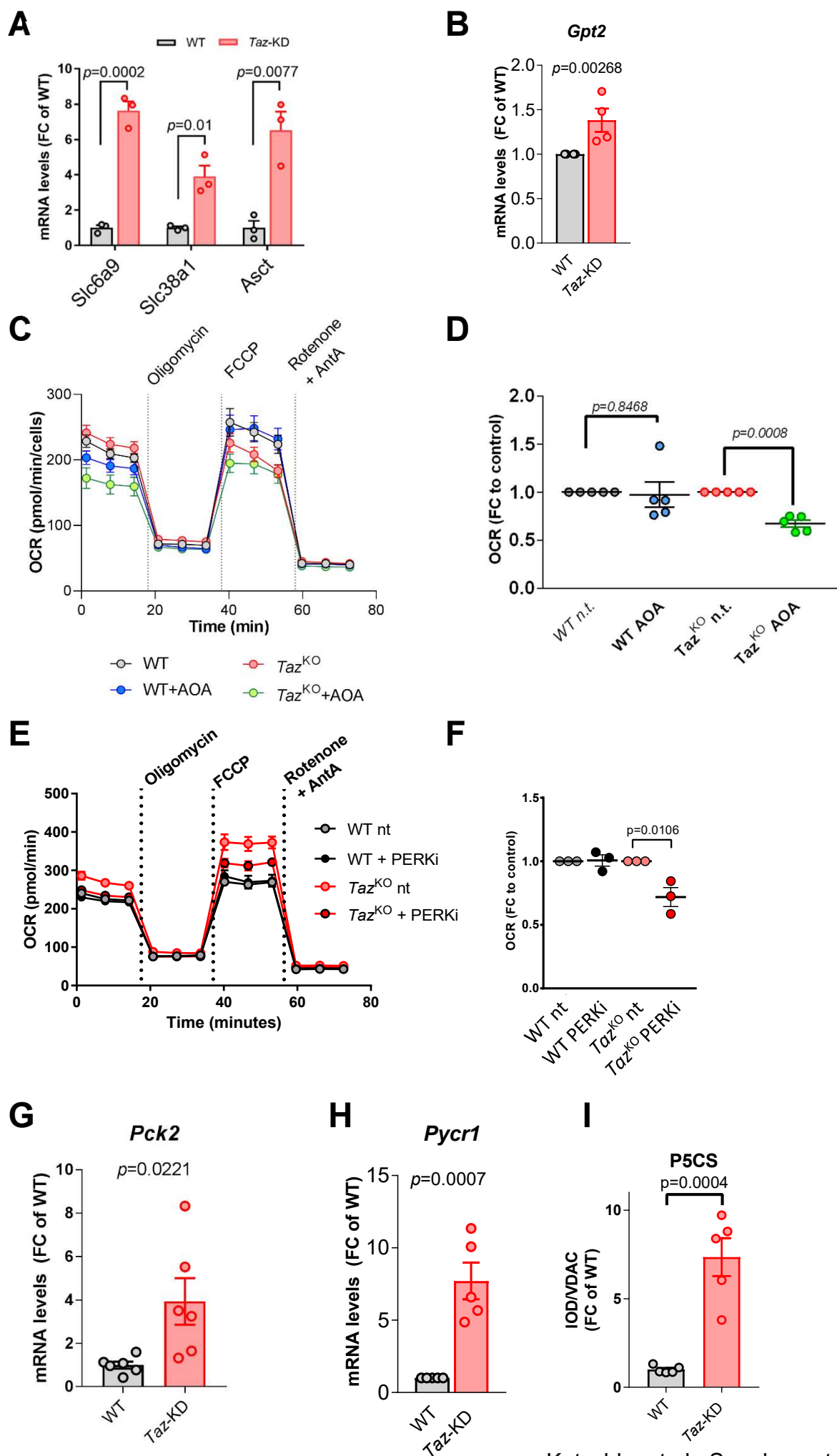

Supplement: Supplementary file 8 — Supplementary file8 (PDF 409 KB) Supplementary Fig. 8 Genes involved in amino acid metabolism in Taz-KD mouse heart. A qPCR analysis of indicated genes related to Gapdh as a control. n=3. B qPCR analysis of Gpt2 related to Gapdh as a control. n=3. C Oxygen consumption rate (OCR) of MEFs supplied with glutamine and treated with aminooxyacetate (AOA) or DMSO. AntA, Antimycin A. D Quantification of five experiments as shown in (C) with basal respiration normalized to n.t. control for both genotypes. n=5. E Respiration (Oxygen consumption rate, OCR) of MEFs supplied in glutamine containing medium and treated with a specific inhibitor of the kinase PERK (PERKi) or DMSO. F Quantification of three experiments as shown in (E) with basal respiration normalized to n.t. control for both genotypes. n=3. G Gene expression of Pck2 related to Gapdh as a control, n=6 for WT and n=5 for Taz-KD. H qPCR analysis of Pycr1 related to Gapdh as a control. n=5. I Quantification of western blot analysis of P5CS (gene product of Aldh18a1), normalized to VDAC as a control (n=5). Data represent mean ± SEM; n-numbers are indicated as numbers of animals. Statistical significance was determined by unpaired Student’s t-test [file 395_2023_1017_MOESM8_ESM.pdf]

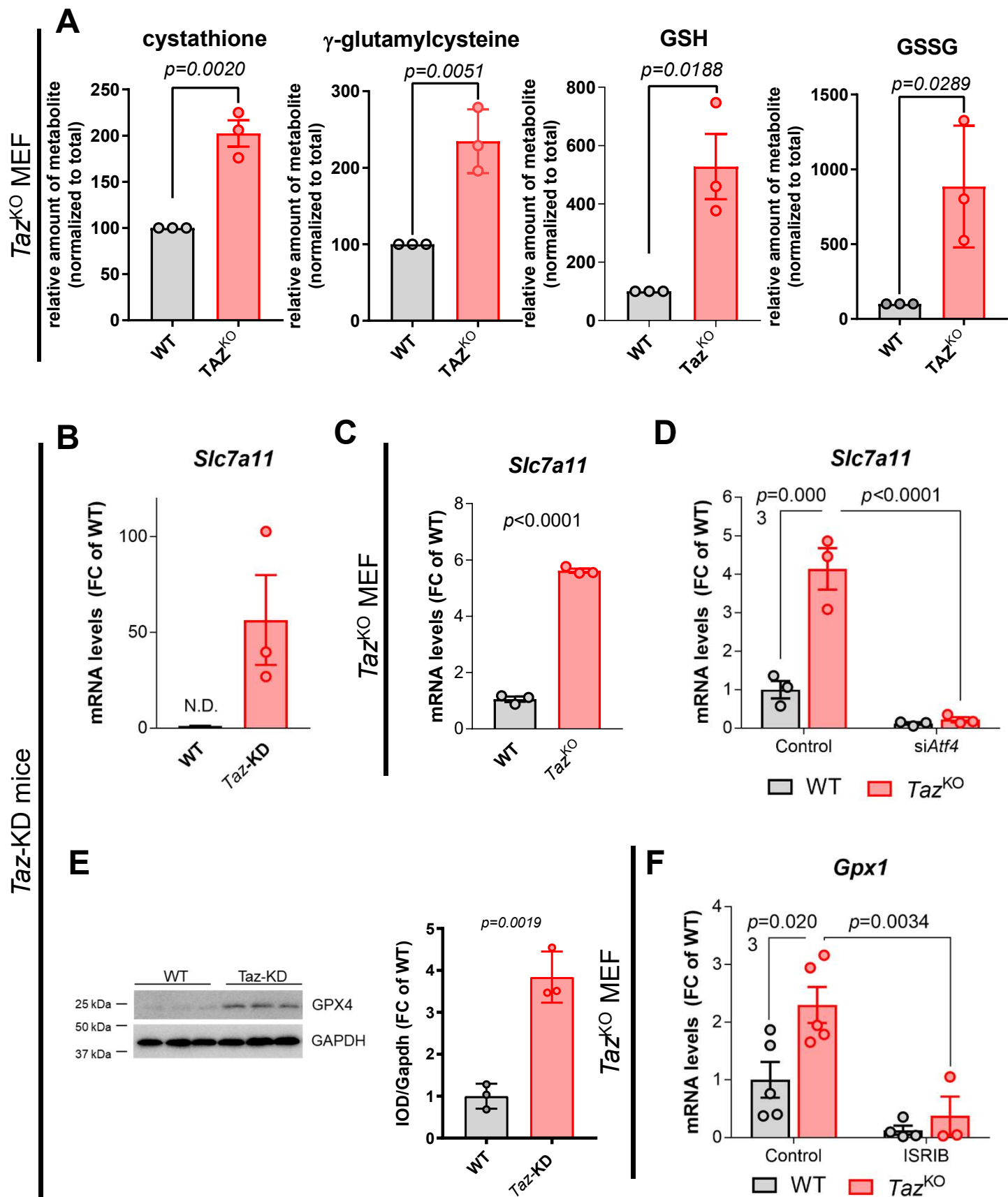

Supplement: Supplementary file 9 — Supplementary file9 (PDF 400 KB) Supplementary Fig. 9 Slc7a11 gene expression in skeletal muscle and MEF cells. A Analysis of indicated metabolites by mass-spectrometry. Relative amounts of metabolites, normalized to total metabolites with WT set to 100%. B qPCR analysis of Slc7a11 gene expression in skeletal muscle tissue from WT and Taz-KD mice displayed as fold change (FC) of WT (n=3, N.D. = not detectable). C qPCR analysis of Slc7a11 gene expression in MEF cells. n=3. D qPCR analysis of Slc7a11 gene expression in MEF cells treated with siRNA against Atf4 or control normalized to Gapdh. n=3. E Western blot analysis of GPX4 and GAPDH in cardiac lysates of Taz-KD vs. WT mice (left panel) and quantification (right panel, n=3). F qPCR analysis of Gpx1 in MEF cells treated with ISRIB or DMSO as control normalized to mS12. n=5. Data represent mean ± SEM; n-numbers represent individual animals in panels B and E, independent cell experiments in A, C, D and D- F. Statistical significance was determined with unpaired Student’s t-test in panel A,C and E. For statistical analysis of panel D and F, a one way ANOVA followed by Tuckey´s multiple comparisons test was performed [file 395_2023_1017_MOESM9_ESM.pdf]
