# Supplementary material for: Activation of the integrated stress response rewires cardiac metabolism in Barth syndrome
Source: Basic Res Cardiol. 2023 Nov 6;118(1):47. doi: 10.1007/s00395-023-01017-x (PMC10628049; doi:10.1007/s00395-023-01017-x)
Supplement: Supplementary file 10 — Supplementary file10 (DOCX 17 KB) [file 395_2023_1017_MOESM10_ESM.docx]

**Supplementary tables for transcriptome analysis of cardiac tissue and skeletal muscle from Taz-KD mice: Usually if accepted some editor/reviewers would ask the authors to upload the raw sequencing data to GEO (https://www.ncbi.nlm.nih.gov/geo/) for review.**

**Suppl. Table 1.** Oligonucleotides

| **Target genes in mus musculus** | **Primer sequence 5´🡪 3´** |
| --- | --- |
| ACADVL for | TATCTCTGCCCAGCGACTTT |
| ACADVL rev | TGGGTATGGGAACACCTGAT |
| ASNS for | TACAACCACAAGGCGCTACA |
| ASNS rev | AAGGGCCTGACTCCATAGGT |
| ATF4 for | GCCGGTTTAAGTTGTGTGCT |
| ATF4 rev | CTGGATTCGAGGAATGTGCT |
| CACT for | GGATGAACTTAGCTACCCA |
| CACT rev | ACTGGCAGGAACATCTC |
| CHOP for | TATCTCATCCCCAGGAAACG |
| CHOP rev | GGGCACTGACCACTCTGTTT |
| CPT1b for | GTCGCTTCTTCAAGGTCTGG |
| CPT1b rev | AAGAAAGCAGCACGTTCGAT |
| CPT2 for | ACCTGCTCGCTCAGGATAAA |
| CPT2 rev | TGTCTTCAGAAACCGCACTG |
| GADD45 for | GCTCAACGTAGACCCCGATA |
| GADD45 rev | GTTCGTCACCAGCACACAGT |
| GAPDH for | CATCACTGCCACCCAGAAGACTG |
| GAPDH rev | ATGCCAGTGAGCTTCCCGTTCAG |
| GPT2 for | GTGGCAGCCTTTATCACCAG |
| GPT2 rev | AGCCCAGCAGTTCTCTTCA |
| mS12 for | GAAGCTGCCAAGGCCTTAGA |
| mS12 rev | AACTGCAACCAACCACCTTC |
| MTHFD2 for | CAGAGGAGCTGGAAGTGTTCAA |
| MTHFD2 rev | TGGCTCAGAGTGCTGCTAGTTG |
| PGC1a for | TGATGTGAATGACTTGGATACAGACA |
| PGC1a rev | GCTCATTGTTGTACTGGTTGGATA |
| PPARa for | CCACGAAGCCTACCTGAAGA |
| PPARa rev | GGACCTCTGCCTCTTTGTCT |
| PSAT1 for | CAGGTCAGTGGGAGGCATTC |
| PSAT1 rev | GGCCGCCAGCTTCTCAA |
| PYCR1 for | GCTGGCCCAAGACATCGT |
| PYCR1 rev | CAGTGCCTAGGGAATCATTGC |
| SLC7A11 for | GTCTGCCTGTGGAGTACTGT |
| SLC7A11 rev | ATTACGAGCAGTTCCACCCA |
| TAZ for | GAAGTTGATGCGTTGGACCC |
| TAZ rev | ACCATCTCCTCGACACACAG |
| TMLHE for | TACAGCACACCGAACCCTAA |
| TMLHE rev | CATGAAGACCCAGGAGACGA |
| **Target genes in homo sapiens** | **Primer sequence 5´🡪 3´** |
| ACADVL for | CCTTTGCAACACCCAGTA |
| ACADVL rev | CCACAGTCTCCCCAGAT |
| ASNS for | GTTGCTGCCACTCTGTTGAA |
| ASNS rev | AGAGCCTGAATGCCTTCCTC |
| CACT for | TACAGATTCAGGCTTCTTCAG |
| CACT rev | CTCACTGACCCTCTTTCC |
| CPT1b for | TCAGCGGTACCTAGAGTC |
| CPT1b rev | TAGTTGCTGTTCACCATGAG |
| CPT2 for | CTGTAGCACTGCCGCATTCA |
| CPT2 rev | AGAGCAAACAAGTGTCGGTCAA |
| L28 for | GCAATTCCTTCCGCTACAAC |
| L28 rev | TGTTCTTGCGGATCATGTGT |
| MTHFD2 for | GAGGTGATGCCACTGTT |
| MTHFD2 rev | ACTCCTTCAAAATCCACATC |
| PYCR1 for | AAGCCACACATCATCCCCTT |
| PYCR1 rev | ACGACTGGAGTGTTGGTCAT |

**Suppl. Table 2.** List of antibodies

| **Primary antibodies** | | |
| --- | --- | --- |
| **Name** | **Producer** | **Identifier** |
| ACADVL | Proteintech | 14527-1-AP |
| Aldh18a1 | Sigma | HPA012604 |
| CACT | Proteintech | 19363-1-AP |
| CoxIV-I | Non-commercial |  |
| Cox5a | Non-commercial |  |
| CPT1b | Abcam | ab134988 |
| CPT2 | Thermo Fisher | PA5-12217 |
| eIF2a | Cell Signaling | #9722 |
| GAC | Proteintech | 19958-1-AP |
| GAPDH | Sigma-Aldrich | MAB374 |
| GPT2 | Proteintech | 167577-1-AP |
| MTHFD2 | Proteintech | 12270-1-AP |
| P5CS (Aldh18a1) | Sigma | HPA012604 |
| P-eIF2a | Cell Signaling | #9721 |
| Perk | Cell Signaling | #3192 |
| P-Perk | Cell Signalling | #3179 |
| PSAT1 | Proteintech | 1051-1-AP |
| Slc7a11 | Thermo Fisher | PA1-16893 |
| Tim23 | non commercial |  |
| TMLHE | Proteintech | 16621-1-AP |
| TUBULIN | Abcam | ab6046 |
| VDAC | non commercial |  |
| **Secondary antibodies** | | |
| **Name** | **Producer** | **Identifier** |
| anti-mouse | Jackson ImmunoReasearch | 115-035-146 |
| anti-rabbit | Jackson ImmunoReasearch | 111-035-144 |

**Suppl. Table 3.** List of siRNAs

| **target gene** | **siRNA sequences** |
| --- | --- |
| ATF4 | CCAUCUCCCAGAAAGUUUA dTdT |
| GADD45 | GGAUCCUGCCUUAAGUCAA dTdT  GAUCCAUUUCACCCUCAUC dTdT  GACGAACCCACAUUCAUCA dTdT |
| GPT2 | CAGCUUGGGAUCUUACAGU dTdT  GACAUGUUCUACUGCAUGA dTdT  GAUGUGGCAGCCUUUAUCA dTdT |
